# C9-ALS-Associated Proline-Arginine Dipeptide Repeat Protein Induces Activation of NLRP3 Inflammasome of HMC3 Microglia Cells by Binding of Complement Component 1 Q Subcomponent-Binding Protein (C1QBP), and Syringin Prevents This Effect

**DOI:** 10.3390/cells11193128

**Published:** 2022-10-05

**Authors:** Ru-Huei Fu, Chia-Wen Tsai, Shao-Chih Chiu, Shih-Ping Liu, Yu-Ting Chiang, Yun-Hua Kuo, Woei-Cherng Shyu, Shinn-Zong Lin

**Affiliations:** 1 Graduate Institute of Biomedical Sciences, China Medical University, Taichung 40402, Taiwan; 2 Translational Medicine Research Center, China Medical University Hospital, Taichung 40447, Taiwan; 3 Department of Nutrition, China Medical University, Taichung 40402, Taiwan; 4 Buddhist Tzu Chi Bioinnovation Center, Tzu Chi Foundation, Hualien 97002, Taiwan; 5 Department of Neurosurgery, Buddhist Tzu Chi General Hospital, Hualien 97002, Taiwan

**Keywords:** amyotrophic lateral sclerosis (ALS), *C9orf72*, proline-arginine dipeptide repeat protein (PR-DPR), HMC3 microglia cell, NLRP3 inflammasome, mouse motor neuron NSC34 cell, complement component 1 q subcomponent-binding protein (C1QBP), syringin

## Abstract

Amyotrophic lateral sclerosis (ALS) is a fatal disease in which motor neurons gradually degenerate. The mutation of the *C9orf72* gene is the main genetic cause of ALS (C9-ALS). One of its specific pathological features is the production of proline-arginine (PR) dipeptide repeat protein (DPR). In this study, we developed a PR-DPR (PR_50_)-expressing human HMC3 microglial cell model. We found that PR_50_ mainly aggregates into spots in the nucleus and induces significant NLRP3 inflammasome activity. Moreover, mouse NSC-34 motor neuron cells treated with a conditional medium of PR_50_-expressing HMC3 cells (PR-CM) caused cell damage and apoptosis activity. However, R_50_-expressing HMC cells treated with MCC950 (an NLRP3 inhibitor) reversed this result. Furthermore, we identified complement component 1 q subcomponent-binding protein (C1QBP) as one of the interaction partners of PR_50_. The downregulation of C1QBP in HMC3 cells induces NLRP3 inflammasome activity similar to PR_50_ expression. Finally, we found that syringin can block the interaction between PR_50_ and C1QBP, and effectively reduce the PR_50_-induced NLRP3 inflammasome activity in HMC3 cells. This improves the apoptosis of NSC-34 cells caused by PR-CM. This study is the first to link PR_50_, C1QBP, and NLRP3 inflammasome activity in microglia and develop potential therapeutic strategies for syringin intervention in C9-ALS.

## 1. Introduction

Amyotrophic lateral sclerosis (ALS) is a progressive, fatal, degenerative disease of the motor neurons that control voluntary muscles [1]. Symptoms begin with muscle stiffness and atrophy, leading to paralysis and death from respiratory failure in most patients within a few years. The cause of most ALS is unknown and currently there is no effective treatment [2]. Therefore, it is imperative to analyze the pathogenic mechanism of ALS and develop effective therapeutic strategies. Mutations in the human chromosome 9 open reading frame 72 (*C9orf72*) gene are the most common form of hereditary ALS (C9-ALS) and are also present in some patients with a sporadic form. This major genetic cause is a GGGGCC (G_4_C_2_) hexanucleotide repeat expansion (HRE) in intron 1 of *C9orf72* [3]. In motor neurons, specific pathological features have been linked to C9-ALS, including a decreased expression of mRNA and proteins of the *C9orf72* (loss of function), the generation of toxic RNA and dipeptide repeat proteins (DPRs) (gain of function), and their downstream effects [4,5]. DPRs produced by unconventional repeat-associated non-ATG translation (RAN) contribute to the toxicity of c9-ALS [6]. Among the five DPRs, poly proline-arginine (PR-DPR) and poly glycine-arginine (GR-DPR), which contain arginine, are more toxic than the other DPRs. Poly-PR is reported to be the most toxic [6].

Previous studies have revealed that PR-DPR forms toxic nuclear aggregates in neurons [7], causing neurodegeneration in *Drosophila* [8] and motor neuron death in mice [9], resulting in cerebellum and spinal cord-related motor imbalances and inflammation. The possible reasons for the neurotoxicity of PR-DPR are as follows. (1) The ribosome biogenesis of nuclei is blocked [10,11,12]. (2) The mechanism of nonsense-mediated mRNA decay is destroyed [13]. (3) The stress-induced RNA editing activity is inhibited [14]. (4) Mis-splicing occurs [15]. (5) The accumulation of RNA-binding protein Staufen increases [16]. (6) The expression of TAR DNA-binding protein 43 is deregulated and increases the formation of the paraspeckle [17]. (7) The transcription factor p53 is activated and downstream events are induced, including the puma transcriptional program [18]. (8) The repair pathway of double-strand DNA breaks is damaged [19]. (9) Karyopherin-mediated cargo loading and nuclear import/export are disturbed [20,21]. (10) Protein translation is hindered [22,23]. (11) The cell-autonomous excitotoxicity of glutamatergic neurons is promoted [24]. (12) Sustained Nav1.2/β4 sodium channel complex activity is enhanced and leads to the hyperexcitability of cortical motor neurons [25]. (13) ER stress is induced [26]. (14) Molecular chaperones are isolated, resulting in impaired protein folding [27]. (15) The capability of microtubule-based cargo transport decreases [28]. Interestingly, the asymmetric dimethylation of PR-DPR by Type I PRMT (arginine methyltransferases) plays an important role in PR-DPR cytotoxicity [29].

C9-ALS causes the abnormal activation of the immune system [30,31,32,33]. The deficiency of the *C9orf72* gene causes gut bacteria-induced systemic and neuropathic inflammation in a mouse model of C9-ALS [34] and fails to suppress the stimulator of interferon genes (STING)-induced type I interferon-mediated inflammation [35]. Additionally, a C9-ALS mouse model expressing high levels of PR-DPR exhibits inflammation of the cerebellum and spinal cord, leading to motor dysfunction and neurodegeneration [9]. Although motor neurons are the major cells influencing ALS, some evidence has lately suggested that microglia in the niche also play a key role in the neurodegeneration of ALS [36]. In the microglial, activation of NOD-, LRR-, and pyrin domain-containing 3 (NLRP3) inflammasomes are believed to have a crucial role in neuroinflammation during neurodegeneration [37,38]. NLRP3 is an intracellular sensor that recognizes pathogenic signals, environmental stimuli, and endogenous damage signals, leading to the development and activation of the NLRP3 inflammasomes [39]. The activation of classical NLRP3 inflammasomes involves two stages: priming and activation. The priming stage begins with the recognition of extracellular molecules, including pathogen-associated molecular patterns (PAMPs) or damage-associated molecular patterns (DAMPs) by pattern recognition receptors, such as toll-like receptors and then the induction of the activity of transcription regulators of nuclear factor-κB (NF-κB). Finally, NF-κB binds to the κB site in the promoter region of the target gene NLRP3, pro-interleukin (IL)-1β and IL-18 to upregulate their expression [40]. Moreover, priming also causes NLRP3 post-translational modifications, such as phosphorylation and ubiquitination, to support the inflammasome assembly. During the activation phase, a broad range of factors, including PAMPs and DAMPs (ATP, nigericin, and cholesterol crystals, etc.), induce NLRP3 oligomerization and recruit the adaptor protein apoptosis-associated speck-like protein containing a CARD (ASC), which then interacts with the cysteine protease caspase-1 to form a complex called the inflammasome. Next, the inflammasome triggers the self-cleavage and the activation of pro caspase-1. The last activated caspase-1 converts pro-IL-1β and pro-IL-18 to the mature active forms [41]. Meanwhile, mature caspase-1 induces the cleavage of the N-terminal fragment of gasdermin-D (GSDMD) to form lytic pores, which promote the release of mature IL-1β and IL-18 and promote pyroptotic-cell death. Pathogenic protein aggregates, such as α-synuclein, initiate NLRP3 activation in the microglial cells, activating ASC, caspase-1, and the secretion of proinflammatory cytokines IL-1β and IL-18, and then contribute to the disease progression of Parkinson’s disease [42]. Recent research also indicated that caspase-1 can further lead to caspase-3 cleavage and activated apoptosis [43]. Studies have also shown that human monocytes can form the classic NLRP3 inflammasome without priming. For example, the NLRP3 activator nigericin causes NLRP3 inflammasome assembly and pro IL-18 processing and release without priming. In the mouse SOD1^G93A^ model of ALS, Deora et al. demonstrated that pathological SOD1^G93A^ proteins activated the NLRP3 inflammasomes of microglia [44]. Although this evidence suggests that the NLRP3 inflammasome is a potential therapeutic target for ALS, whether the microglia similarly sense PR-DPR stimulation and initiate NLRP3 inflammasome activation and the detailed mechanisms in the pathogenesis of C9-ALS remain unclear.

In this study, we used a cellular model to confirm that endogenous PR-DPR affects the activity of the NLRP3 inflammasomes in microglia and leads to motor neuron apoptosis. Moreover, we also elucidated that PR-DPR activates intracellular NLRP3 inflammasome activity by directly binding and inhibiting the function of complement component 1 q subcomponent-binding protein (C1QBP). Finally, we found that siringin can block the interaction between PR-DPR and component 1 Q subcomponent-binding protein (C1QBP), which may be used for therapeutic intervention in C9-ALS in the future.

## 2. Materials and Methods

### 2.1. Cell Culture and Chemicals

Culture media and related regents were purchased from Gibco (Thermo Fisher Scientific, Waltham, MA, USA). Chemicals were acquired from Sigma-Aldrich (St. Louis, MO, USA) unless otherwise stated. HMC3 human microglial cells were a generous gift from Shao-Chih Chiu (China Medical University, Taichung, Taiwan). Cells were cultured in MEM with 10% fetal bovine serum (FBS), 1 mM sodium pyruvate, 1500 mg/L sodium bicarbonate, 2 mM L-glutamine, 100 I.U./mL penicillin, and 100 μg/mL streptomycin, and were maintained at 37 °C in an incubator under humidified atmosphere of 5% CO_2_. NSC-34 mouse motor neuron cell lines were a generous gift from Shinn-Zong Lin (Tzu Chi University, Hualien, Taiwan). Cells were cultured in DMEM with 10% fetal bovine serum (FBS), 100 I.U./mL penicillin, and 100 μg/mL streptomycin, and were maintained at 37 °C in an incubator under humidified atmosphere of 5% CO_2_.

### 2.2. Plasmid Construction and Transfection

The DNA fragments of PR_50_ and C1QBP were generated by gene synthesis (Genewiz, NJ, USA). PR_50_ fragment is digested by restriction enzyme and inserted into the pcDNA 3.1/myc-His vector (Invitrogen, ThermoFisher Scientific, Carlsbad, CA, USA), pGBKT7 vector (Clontech, Mountain View, CA, USA), pGADT7 vector (Clontech), pCMV-Myc (Clontech), or pCMV-HA (Clontech). Finally, according to the manufacturer’s instructions, Lipofectamine 2000 reagent (Invitrogen) was used to transfect the plasmid into cell lines. Empty vectors were used as the control group. The transfected cells were selected using G418 (1.5 mg/mL). The cells and conditional media were collected for various analyses and experiments.

### 2.3. Immunofluorescence Analysis

Cellular samples grown on poly-L-lysine-coated coverslips were washed and fixed with 4% paraformaldehyde at room temperature for 10 min and then incubated with 0.2% Triton X-100 for 10 min. Next, the samples were soaked in a solution containing 1% BSA and 22.52 mg/mL glycine [dissolved in PBST (PBS + 0.1% Tween 20)] for 30 min. Primary antibody was added and allowed to react overnight at 4 °C. The next day, the sample was washed five times and placed in PBST containing 1% BSA. Goat anti-mouse IgG secondary antibody-Alexa Fluor 488 and goat anti-rabbit IgG secondary antibody-Alexa Fluor 568 (purchased from Invitrogen) were added simultaneously, and the sample reacted at 25 °C for 1 h. Finally, the sample was washed, the nuclei were stained with 4,6-diamidino-2-phenylindole (DAPI), and the fluorescence was detected using a Zeiss Axio Imager A1 fluorescence microscope (Carl Zeiss MicroImaging GmbH, Göttingen, Germany). Myc, C1QBP and NF-κB p65 antibody was purchased from Cell Signaling Technology (Beverly, MA, USA).

### 2.4. Preparation of Nuclear Extract and NF-κB (p65) Transcription Factor Assay

Cell samples were washed and collected. The pellet was added to hypotonic buffer (10 mmol/L HEPES, 1 mmol/L MgCl_2_, 10 mmol/L KCl, 0.5 mmol/L DTT, 1 mmol/L EDTA, 20 μg/mL aprotinin, 4 μg/mL leupeptin, 0.2 mmol/L phenylmethylsulfonyl fluoride, and 0.5% Nonidet P-40), and stood on ice for 20 min. Afterwards, crude nuclei were acquired after centrifugation at 6000× *g* for 20 min and pellets were resuspended in hypertonic buffer (10 mmol/L HEPES, 1 mmol/L MgCl_2_, 400 mmol/L KCl, 0.5 mmol/L DTT, 1 mmol/L EDTA, 20 μg/mL aprotinin, 4 μg/mL leupeptin, 0.2 mmol/L phenylmethylsulfonyl fluoride, and 25% glycerol). After incubating for an additional 30 min, the nuclear extracts were obtained by centrifugation at 10,000× *g* for 20 min and were frozen at −80 °C. In the NFκB p65 assay, we used NFκB p65 EZ-TFA Transcription Factor Assay Colorimetric Kit (Millipore, Temecula, CA, USA). Briefly, the NFκB response element (a double stranded DNA sequence) was immobilized onto the bottom of the wells of a 96-well plate. NFκB of nuclear extract bound to the NFκB response element, and was identified using an anti NFκB p65 antibody. A secondary antibody conjugated to HRP was added and then it reacted with the substrate to provide a colorimetric readout at 450 nm.

### 2.5. Western Blotting

Cells were washed with cold PBS and then lysed in RIPA buffer (150 mM NaCl, 25 mM Tris-HCl, 1% Triton X-100, 10% glycerol, 2 mM EDTA, 1 μg/mL aprotinin, 1 μg/mL leupeptin, 1 mM PMSF, and phosphatase inhibitor). Next, cell lysates were centrifuged at 14,000× *g* for 20 min at 4 °C. The supernatant was obtained, and the protein concentration was determined by Comassie Plus Protein Assay Reagent Kit (Pierce, Rockford, IL, USA). Fifty grams of cellular extract were boiled with the sodium dodecyl sulfate (SDS)-containing sample buffer for 10 min and separated by 7.5–12.5% SDS-polyacrylamide gel electrophoresis (SDS-PAGE). The samples were then transferred to a polyvinylidene fluoride (PVDF) membrane. After reacting with the primary antibody overnight, the protein was detected using horseradish peroxidase (HRP)-conjugated secondary antibody (PerkinElmer Inc., Boston, MA, USA). Finally, the position and intensity of the specific protein was determined by the Amersham enhanced chemiluminescence system (Amersham Biosciences, Piscataway, NJ, USA) and BioSpectrum imaging system (UVP, Upland, CA, USA). NLPR3, ASC, caspase 1, cleaved caspase 1, IL-1β, cleaved IL-1β, IL-18, cleaved IL-18, caspase-9, cleaved caspase-9, caspase-7, cleaved caspase-7, caspase-3, cleaved caspase-3, poly-ADP ribose polymerase (PARP), and cleaved PARP antibodies were from Cell Signaling Technology. Monoclonal antibodies against GAPDH were from Santa Cruz Biotechnology, Inc. (Santa Cruz, CA, USA). HRP-conjugated goat anti-rabbit and HRP-conjugated goat anti-mouse secondary antibodies were obtained from PerkinElmer, Inc. (Boston, MA, USA).

### 2.6. Conditioned Medium Collection and ELISA

The conditioned medium of HMC3 cells was collected and centrifuged at 1000× *g* for 5 min. Then, the supernatant was passed through a 0.22 µm filter to eliminate smaller debris and was placed on dry ice for snap-freezing and stored at −80 °C until use. Human IL-1 β ELISA Kit (invitrogen), IL-18 ELISA Kit (invitrogen), and c-Myc ELISA Kit (invitrogen) were used according to manufacturer’s protocol. 50 µL of the conditioned medium was used in each well of ELISA plates to detect. The plates were then analyzed on a SpectraMax M2 Microplate Reader (Molecular Devices, Silicon Valley, CA, USA) and the absorbance was read at 450 nm.

### 2.7. Lactate Dehydrogenase Assay

The release of the intracellular enzyme lactate dehydrogenase (LDH) into the medium is used as a quantitative measurement of NSC34 cell viability. NSC-34 cells were treated with a conditional medium of PR_50_-expressing HMC3 cells at 1/10, 1/5, or 1/2 medium volume ratios for 24 h and then the culture medium was measured by LDH-cytotoxicity assay kit (Sigma-Aldrich) according to the manufacturer’s instructions.

### 2.8. In Situ TUNEL Assay

Cell apoptosis was calculated with a Click-iT™ Plus TUNEL Assay kit for in situ apoptosis detection (Alexa Fluor™ 488 dye) according to manufacturer’s directions (invitrogen). Briefly, cells in coverslips were washed and fixed in 4% paraformaldehyde at room temperature for 15 min. Then we removed the fixative, added the permeabilization reagent (0.25% Triton X-100 in PBS), and incubated the samples for 20 min at room temperature. Next, samples were preincubated in Tdt reaction buffer for 10 min at 37 °C then for a further 60 min at 37 °C with Tdt reaction mixture containing Tdt enzyme, EdUTP, and Tdt reaction buffer. After that, samples were rinsed in 3%BSA/PBS and were detected with the Click-iT™ plus TUNEL reaction cocktail for 30 min at 37 °C in the dark. Finally, samples were rinsed in 3%BSA/PBS and imaging was conducted.

### 2.9. Measurement of Mitochondrial Membrane Potential (MMP)

For measurement of MMP of NSC 34 cells, we employed the 3,3′-dihexyloxacarbocyanine iodide (DiOC6) method. The cells were washed twice with PBS and exposed to DiCO6 dye (1 μM) in a fresh medium. Thirty minutes later, the MMP was determined by use of an Axio Observer inverted fluorescence microscope (Carl Zeiss MicroImaging GmbH, Göttingen, Germany) and the intensity of the fluorescence image was quantified using ImageJ software (version 1.50, National Institutes of Health).

### 2.10. Apoptosis Assay by Flow Cytometry

We used the FITC Annexin-V Apoptosis Detection Kit I (BD Biosciences Pharmingen, San Diego, CA, USA) to implement apoptosis analysis according to the manufacturer’s instructions. The cells were collected and resuspended in 100 μL of 1× binding buffer [10 mM HEPES/NaOH (pH 7.4), 140 mM NaCl, and 2.5 mM CaCl_2_]. Then annexin-V FITC and PI were added to stain for 15 min in the dark. Next, 400 μL of 1× binding buffer was added and the apoptosis rate was immediately determined using a BD LSRII flow cytometer (Becton Dickinson, Heidelberg, Germany). The cell collection gate for each sample contained at least 10,000 events. Among them, Q2 is a late apoptotic cell, Q4 is an early apoptotic cell, Q3 is a live cell, and Q1 is a dead cell. The apoptosis rate = (Q2 + Q4)/(Q1 + Q2 + Q3 + Q4) × 100%.

### 2.11. Treatment of Inhibitors of NLRP3 Inflammasomes

A diarylsulfonylurea-containing compound, MCC950, is known to be one of the most potent and selective inhibitors of NLRP3 inflammasome. MCC950 was purchased from APExBIO (Houston, USA). PR_50_-overexpressing HMC3 cells were incubated in fresh medium with MCC950 (1 μM) for 24 h, then various analyses were performed.

### 2.12. Yeast Two-Hybrid Library Screening

First, we constructed Matchmaker cDNA library of human HMC3 cells according to the operation manual (Clontech, Mountain. View, CA, USA). In a Matchmaker GAL4-based two-hybrid assay (Clontech, Mountain. View, CA, USA), the bait (PR_50_) was cloned into a yeast GAL4 DNA-binding domain vector (DNA-BD, pGBKT7) and transformed into *Saccharomyces cerevisiae* host strain AH109, while the prey of cDNA library of human HMC3 cells was expressed as fusions to the GAL4 activation domain (AD, pGADT7 vector) in *Saccharomyces cerevisiae* host strain Y187. We mated the library strain with the bait strain to create diploids. When bait and prey fusion proteins interacted, the DNA-BD and AD were brought into proximity to activate the transcription of four reporter genes (*ADE2*, *HIS3*, *MEL1*, and *LacZ*). According to the manufacturer’s instructions, the interaction of PR_50_ with the candidate protein is determined based on SD/-Ade/-His/-Leu/-Trp X-α-gal plate or colony lift assay. To identify the gene responsible for the positive interaction, we rescued the plasmid from yeast cells grown on SD/-Ade/-His/-Leu using Zymoprep™ Yeast Plasmid Miniprep I (Zymo Research Corporation, Irvine, CA, USA). The prey insert can be identified by sequencing.

### 2.13. Yeast Two-Hybrid Assays

In a Matchmaker GAL4-based two-hybrid assay (Clontech), a bait protein PR_50_ is expressed as a fusion to the Gal4 DNA-binding domain (DNA-BD, pGBKT7 vector), while a prey protein C1QBP (or fragments of CIQBP) is expressed as a fusion to the Gal4 activation domain (AD, pGADT7 vector). The experimental procedure was as described in 2.12. The specific association between PR_50_ and C1QBP (or fragments of CIQBP) was further confirmed by reverse yeast two-hybrid assay.

### 2.14. Co-Immunoprecipitation Analysis

In co-immunoprecipitation analysis, PR_50_ was subcloned into the pCMV-Myc vector (Clontech, Mountain. View, CA, USA) and C1QBP was subcloned into the pCMV-HA vector (Clontech, Mountain. View, CA, USA). Then, both plasmids were co-transfected into 293 T cells using Lipofectamine 2000 transfection reagent (Invitrogen) according to the manufacturer’s instructions. After 24 h, co-transfected cells were lysed in EBC buffer [50 mM Tris-HCl (pH 8.0), 120 mM NaCl, 0.5% NP-40, 1 mM PMSF, 1 μg/mL aprotinin and 1 μg/mL leupeptin], and the soluble supernatant was collected by centrifugation at 14,000× *g* for 5 min at 4 °C. The supernatant was precleared by protein G-Sepharose beads, and then immunoprecipitated with Myc-Tag rabbit monoclonal antibody (Cell Signaling Technology) or a normal rabbit immunoglobulin G (NRIgG) for 2 h at 4 °C, and incubated with protein G-Sepharose beads for an additional 1 h. Immunoprecipitates were then washed three times with EBC buffer and twice with phosphate-buffered saline (PBS). The samples were detected by Western blotting using HA-Tag mouse monoclonal antibody (Cell Signaling Technology). For reverse immunoprecipitation, the cell lysates were immunoprecipitated with HA-Tag mouse monoclonal antibody and analyzed by Western blotting with Myc-tag rabbit monoclonal antibody. Moreover, C1QBP was subcloned into the pCMV-Myc vector and PR_50_ was subcloned into the pCMV-HA vector. Co-immunoprecipitation was performed as described above.

### 2.15. RNA Interference Technique

HMC3 cells were plated on 6-well culture plants at a density of 2.0 × 10^5^ cells per well. When 70% confluence was reached, the cells were transfected with C1QBP siRNA (75 nM) or nontargeting control siRNA using Lipofectamine 2000 transfection reagent (Invitrogen) according to the manufacturer’s protocol. The sense sequences of C1QBP siRNA were as follows: (1) 5′-GAAGGCCCUUGUGUUGGACUGUCAU-3′ and (2) 5′-ACUGGCGAGUCUGAAUGGAAGGAUA-3′. After 24 h, cell lysates and culture medium were collected and various experiments were performed. 

### 2.16. Syringin (SRG) Treatment and Yeast Two-Hybrid-Based Growth Assay

Synthesized syringin (SRG, mol. wt. 372.4, 98% purity) was purchased from Rainbow Biotechnology Co. Ltd. (Shilin, Taipei, Taiwan), dissolved in dimethyl sulfoxide (DMSO) to 1 M, and stored at −20 °C as a master stock solution. Yeast diploids carrying Gal 4 BD-/Gal 4 AD-, Gal 4 BD-p53/Gal 4 AD-T, or Gal 4 BD-PR_50_/Gal 4 AD-C3 (C1QBP) were grown overnight at 30 °C in the broth of SD/-Leu/-Trp until they reached log or mid-log phase. In the first experiment, diploid culture was normalized for OD_600_, serially diluted, and spotted onto solid media of SD/-Leu/-Trp or SD/-Ade/-His/-Leu/-Trp plates with serially diluting SRG using pipetman (10 μL), and grown at 30 °C for 3 days. In the other experiment, diploid culture was normalized for OD_600_, and grown in the broth of SD/-Leu/-Trp or SD/-Ade/-His/-Leu/-Trp/X-α-gal with serially diluting SRG. Then, we recorded the OD value every 12 h for 48 h.

### 2.17. Cytotoxicity Analysis and Treatment of Syringin (SRG) in HMC3 Cells

In the cytotoxicity analysis, HMC3 cells were treated with serial dilutions of syringin (SRG) for 24 h, then cells were washed and replaced with fresh medium. Next, MTT (5 mg/mL) was added, and the cells were incubated at 37 °C. After 2 h, the cells were washed, and the formazan crystals were dissolved in isopropanol. Finally, the absorbance was measured at 570 nm using a SpectraMax M2 Microplate Reader (Molecular Devices). For treating SRG, PR_50_-expressing HMC3 cells were treated with SRG for 24 h, and then cells and conditional mediums were collected and various experiments were performed.

### 2.18. Statistical Analysis

Statistical work was fulfilled using SAS software 9.3 (SAS, Institute. Inc., Cary, NC, USA) Each experiment was performed in triplicate. Data are presented as mean ± standard deviation (SD). We determined statistical significance by employing one-way analysis of variance (ANOVA) and Tukey’s test. The two groups were compared using the student’s *t*-test. *p* values < 0.05 were assumed to show statistical significance.

## 3. Results

### 3.1. Expression of Proline-Arginine (PR)-Dipeptide Repeat Protein (PR_50_) Enhanced Activities of Nuclear Factor kappa B (NF-κB) and NOD-, LRR- and Pyrin Domain-Containing Protein 3 (NLRP3) Inflammasomes in Human HMC3 Microglial Cell

**Figure 1:**
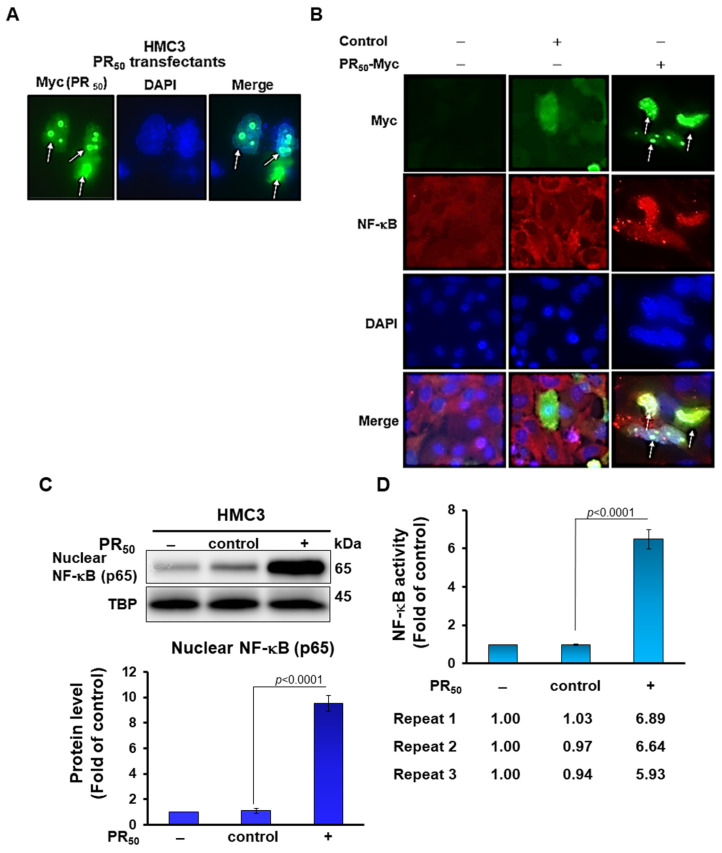

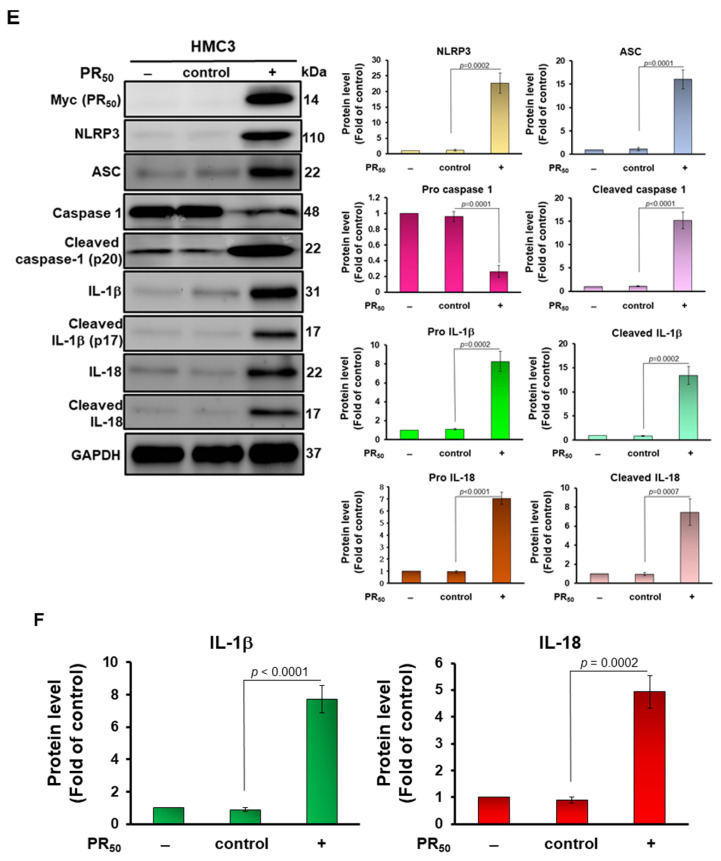

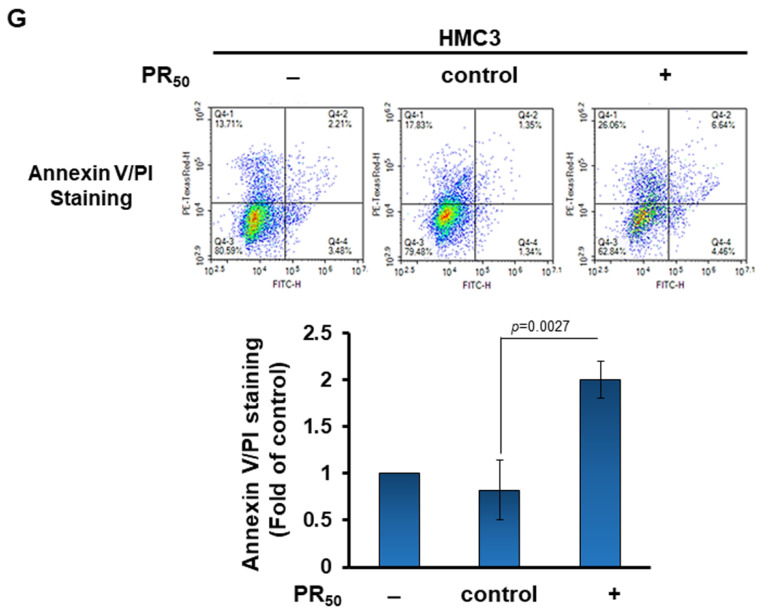
Intracellular expression of PR_50_ induces priming and activation of the NLRP3 inflammasome in human HMC3 microglial cells. HMC3 cells were transfected with a control vector or PR_50_ plasmid for 24 h. (**A**) Immunofluorescence staining of PR_50_ (green) using myc-tag antibody. DAPI was used for nuclear staining. PR_50_ mainly aggregated into spots in the nucleus (white arrows). (**B**) Immunofluorescence staining of NF-κBp65 (red) and Myc-tag (green). Expression of PR_50_ promotes the movement of NF-κB p65 from the cytoplasm to the nucleus (white arrows). DAPI was used for nuclear staining. (**C**) Extract of cell nucleus was analyzed for the level of NF-κB p65 by Western blotting. TBP was used as a loading control. The bottom panel is a quantitative analysis graph using ImageJ software. (**D**) NF-κB p65 transcription factor activity assay showed that expression of PR_50_ promotes NF-κB activity. TBP was used as a loading control for input. This study was performed in triplicate (n = 3). (**E**) Cell lysates were analyzed for the expression of PR_50_ (Myc), NLRP3, ASC, pro caspase-1, cleaved caspase-1 (p20), pro IL-1β, cleaved IL-1β (p17), pro IL-18, and cleaved IL-18 by Western blotting. GAPDH was used as a loading control. The left panel shows a representative set of images, and the right panel shows quantitative analysis graphs using ImageJ software. (**F**) The level of mature IL-1β and mature IL-18 in the conditional medium of all groups were measured by ELISA. (**G**) Apoptotic cell ratio was determined by annexin v-FITC and propidium iodide (PI) staining via flow cytometry (upper panel). Q2 is a late apoptotic cell, Q4 is an early apoptotic cell, Q3 is a live cell, and Q1 is a dead cell. Apoptosis ratio = (Q2 + Q4)/(Q1 + Q2 + Q3 + Q4) × 100% (lower panel).

The pathway of NLRP3 inflammasomes includes a priming step through the activation of the nuclear factor kappa B (NF-κB) and a subsequent activating step to form cleaved caspase-1, cleaved interleukin (mIL)-1β, and cleaved IL-18 (mIL-18) [32]. First, we wanted to determine whether proline-arginine (PR) dipeptide repeat protein (DPR) can stimulate NF-κB and the NLRP3 inflammasome activity of microglia. We transiently transfected and expressed PR-DPR (PR_50_) in the human HMC3 microglial cell line. After transfection for 24 h, we found that PR_50_ mainly aggregates in spots in the nucleus and a small part is scattered in the cytoplasm via an immunofluorescence analysis (Figure 1A). Moreover, the expression of PR_50_ can significantly induce the activity of NF-κB and NLRP3 inflammasomes in HMC3 cells. In the immunofluorescence staining, we found that PR_50_ promoted NF-ΚB (p65) to enter the nucleus (Figure 1B). The Western blotting of nuclear extracts indicated that PR_50_ increases NF-κB (p65) entry into the nucleus compared with the control (empty vector) group (*p* < 0.0001) (Figure 1C). Similarly, PR_50_ augmented NF-κB activation compared with the control (empty vector) group (*p* < 0.0001) by an NF-κB p65 transcription factor activity assay (Figure 1D). Through Western blotting analysis, we found that the expression of PR_50_ can raise the level of NLRP3 (*p* = 0.0002), ASC (*p* = 0.0001), cleaved caspase 1 (p20) (*p* < 0.0001), pro-IL-1β (*p* = 0.0002), cleaved IL-1β (p17) (*p* = 0.0002), pro-IL-18 (*p* < 0.0001), and cleaved IL-18 (*p* = 0.0007) compared with the control group (Figure 1E). ELISA also showed that mature IL-1β (*p* < 0.0001) and mature IL-18 (*p* = 0.0002) level in the conditional medium of PR_50_-expressing HMC3 cells (PR-CM) were augmented compared with the control group (Figure 1F). However, PR_50_ (Myc-Tag) was not detected in the culture media (data not shown). The above data show that the intracellular expression of PR_50_ can promote the activity of NLRP3 inflammasomes in microglia. In addition, we also found that HMC3 cells expressing PR_50_ slightly increased the proportion of apoptotic cells (*p* = 0.0027, Figure 1G).

### 3.2. Conditional Medium of PR-DPR (PR_50_)-Expressing Human HMC3 Microglial Cells Caused the Damage and Apoptosis of Mouse NSC 34 Motor Neuron Cells

**Figure 2:**
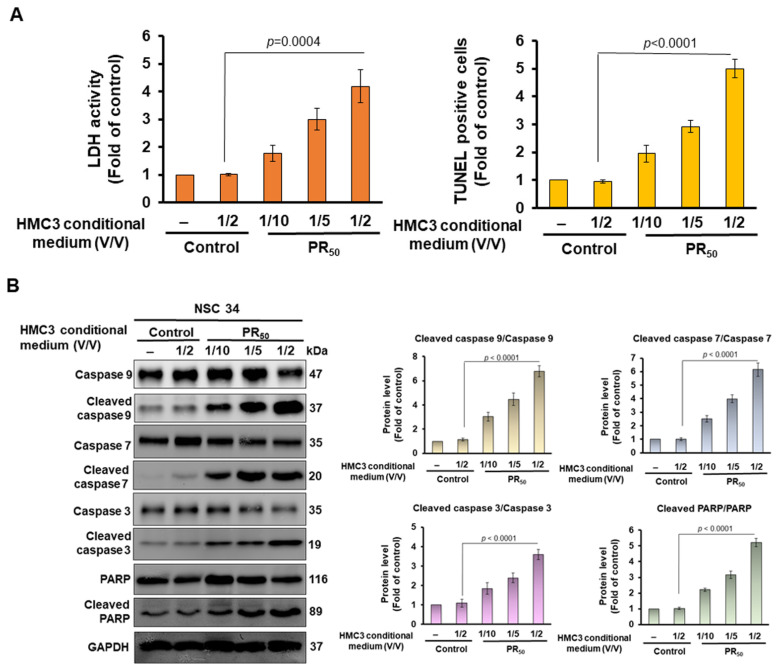

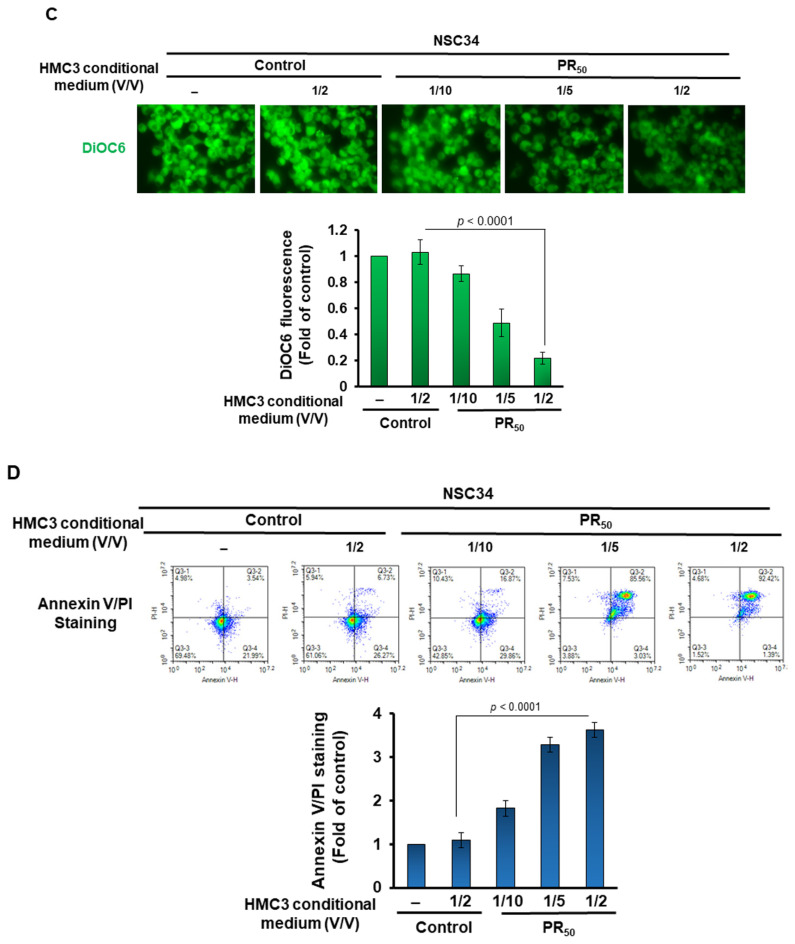
The conditional medium of PR_50_-expressing HMC3 cells (PR-CM) caused damage and apoptosis of mouse NSC-34 motor neuron cells. NSC-34 cells were treated with PR-CM at 1/2 (*v*/*v*), 1/5 (*v*/*v*) and 1/10 (*v*/*v*) volume ratios for 24 h. (**A**) Cell death was measured by lactate dehydrogenase (LDH) release via ELISA and apoptotic cells were determined by TUNEL assay. (**B**) Western blot was used to quantify the levels of apoptotic core proteins (left panel). ImageJ software was used to obtain the signal intensity of the image (right panel). The loaded internal control is GAPDH. (**C**) Mitochondrial membrane potential was determined by DiOC6 staining (green) (upper panel) and the signal intensity of the image was quantified by ImageJ software (lower panel). (**D**) Apoptotic-cell ratio was determined by annexin v-FITC and propidium iodide (PI) staining via flow cytometry (upper panel). Q2 is a late apoptotic cell, Q4 is an early apoptotic cell, Q3 is a live cell, and Q1 is a dead cell. Apoptosis ratio = (Q2 + Q4)/(Q1 + Q2 + Q3 + Q4) × 100% (lower panel).

The activation of the NLRP3 inflammasomes in microglial cells plays a vital role in the establishment of neurodegenerative diseases, including ALS, which cause the degeneration of surrounding motor neurons [45]. The mouse motor neuron-like hybrid-cell line (NSC-34) is a widely used model of mammalian motor neurons [46]. Next, we wanted to determine whether a conditional medium of PR-DPR-expressing human HMC3 microglial cells (PR-CM) can cause motor neuron damage and apoptosis. We treated NSC-34 cell lines with PR-CM at 1/2 (*v*/*v*), 1/5 (*v*/*v*), and 1/10 (*v*/*v*) volume ratios for 24 h. The ELISA showed that PR-CM caused the release of lactate dehydrogenase [in 1/2 (*v*/*v*) group, *p* = 0.0004] and an increase in TUNEL-positive cells [in 1/2 (*v*/*v*) group, *p* < 0.0001] in NSC34 cells in a dose-dependent manner compared with individual control groups (Figure 2A). We also found by the Western blotting analysis that the values of cleaved caspase 9/caspase 9 [in 1/2 (*v*/*v*) group, *p* < 0.0001], cleaved caspase 7/caspase 7 [in 1/2 (*v*/*v*) group, *p* < 0.0001], cleaved caspase 3/caspase 3 [in 1/2 (*v*/*v*) group, *p* < 0.0001], and cleaved PARP/PARP [in 1/2 (*v*/*v*) group, *p* < 0.0001] were increased dose-dependently in the PR-CM group of NSC-34 cells compared with the individual control groups (Figure 2B).

The results of the DiCO6 staining showed that the mitochondrial membrane potential (MMP) of the NSC-34 cells treated with PR-CM was significantly dose-dependently diminished compared with the individual control group [in 1/2 (*v*/*v*) group, *p* < 0.0001] (Figure 2C). To quantify apoptosis, we employed a flow cytometry analysis of annexin-V conjugated to FITC and propidium iodide (PI) staining. As shown in Figure 2D, compared with those in the individual control group, apoptotic cells of NSC-34 were higher in the PR-CM group in a dose-dependent manner [in 1/2 (*v*/*v*) group, *p* < 0.0001]. The above results confirm that PR-CM could promote the damage and apoptosis of motor neurons.

### 3.3. Treatment of PR_50_-Expressing HMC3 Cells with the NLRP3 Inflammasome Inhibitor MCC950 Abolished the Capability That Its Conditional Medium Induces Apoptosis for NSC34 Cells

**Figure 3:**
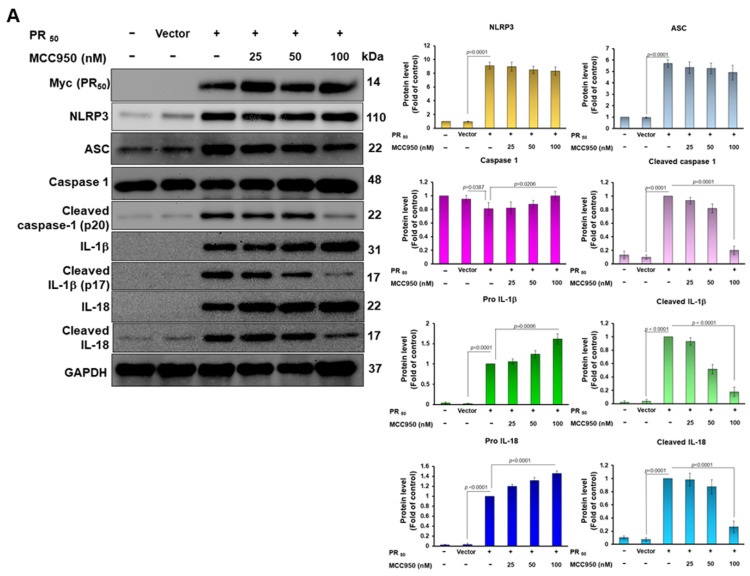

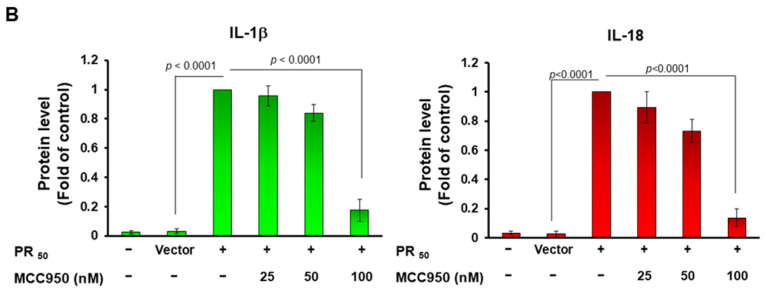
MCC950 inhibited NLRP3 inflammasome activity in PR_50_-expressing HMC3 microglial cells. PR_50_-expressing HMC3 cells were treated with 0, 25, 50, or 100 nM MCC950 for 24 h. (**A**) Cell lysates were analyzed for the expression of PR_50_ (Myc), NLRP3, ASC, pro caspase-1, cleaved caspase-1 (p20), pro IL-1β, cleaved IL-1β (p17), pro IL-18, and cleaved IL-18 by Western blotting. GAPDH was used as an internal loading control. The left panel shows a representative set of images, and the right panel shows quantitative analysis graphs using ImageJ software. (**B**) The level of mature IL-1β and mature IL-18 in the conditional medium were measured by ELISA.

To confirm that NLRP3 inflammasome activity in PR_50_-expressing HMC3 cells is the main cause of inducing apoptosis in NSC34 cells, we treated PR_50_-expressing HMC3 cells with the NLPR3 inflammasome inhibitor MCC950. Compared with the untreated group, the expression of NLRP3 and ASC was not significantly changed in PR_50_-expressing HMC3 cells after treatment with 100 nM MCC950. However, the levels of cleaved caspase 1 (p20) (*p* < 0.0001), cleaved IL-1β (p17) (*p* < 0.0001), and cleaved IL-18/IL-18 (*p* < 0.0001) were significantly diminished (Figure 3A). In the ELISA, we found that the level of mature IL-1β (*p* < 0.0001) and mature IL-18 (*p* < 0.0001) in the conditional medium of the 100 nM MCC950-treated PR50-expressing group were also significantly reduced compared to the untreated control group (Figure 3B). The above data show that MCC950 treatment can effectively prevent PR_50_-induced NLRP3 inflammasome activity in HMC3 microglial cells.

**Figure 4:**
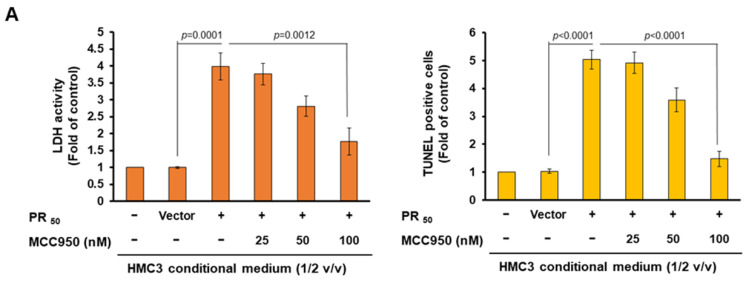

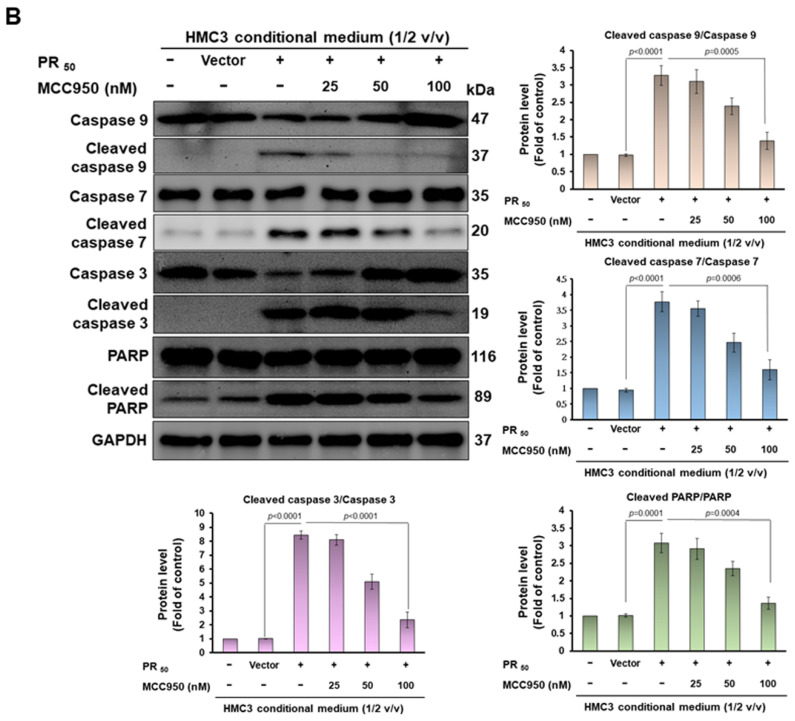
MCC950 treatment abolished the ability that conditioned medium of PR_50_-expressing HMC3 cells (PR_50_-CM) induced apoptosis in the mouse NSC-34 motor neuron cells. NSC-34 cells were treated with conditional medium of 0, 25, 50, or 100 nM MCC950-treated PR_50_-expressing HMC3 cells (PR-CM) at 1/2 (*v*/*v*) volume ratios for 24 h. (**A**) Cell death and apoptosis were measured by lactate dehydrogenase (LDH) release via ELISA and by apoptotic cells number via TUNEL assay, respectively. (**B**) Western blot was used to quantify the levels of apoptotic core proteins (left panel). ImageJ software was used to obtain the signal intensity of the image (right panel). The loaded internal control is GAPDH.

Next, we want to determine whether the conditional medium of PR_50_-expressing HMC3 cells (PR-CM) causing motor neuron death and apoptosis can be blocked by MCC950 treatment. We found that the PR-CM of the 100 nM MCC950-treated group revealed a reduction in lactate dehydrogenase release (*p* = 0.0012) and TUNEL-positive cells (*p* < 0.0001) in NSC34 cells compared with the MCC950 untreated group (Figure 4A), indicating that NSC34 cells recover from damage and apoptosis. We also found by Western blotting that the values of cleaved caspase-9/caspase-9 (*p* = 0.0005), cleaved caspase-7/caspase-7 (*p* = 0.0006), cleaved caspase-3/caspase-3 (*p* < 0.0001) and cleaved PARP/PARP (*p* = 0.0004) were significantly reduced in the 100 nM MCC950-treated group compared with the untreated group (Figure 4B). The above experiments showed that the PR-CM induced motor neuron apoptosis mainly through NLPR3 inflammasome activity-associated IL-1β and IL-18.

### 3.4. Complement Component 1 Q Subcomponent-Binding Protein (C1QBP) Is a Candidate Interaction Partner of PR-DPR (PR_50_) in HMC3 Microglial Cells

**Figure 5:**
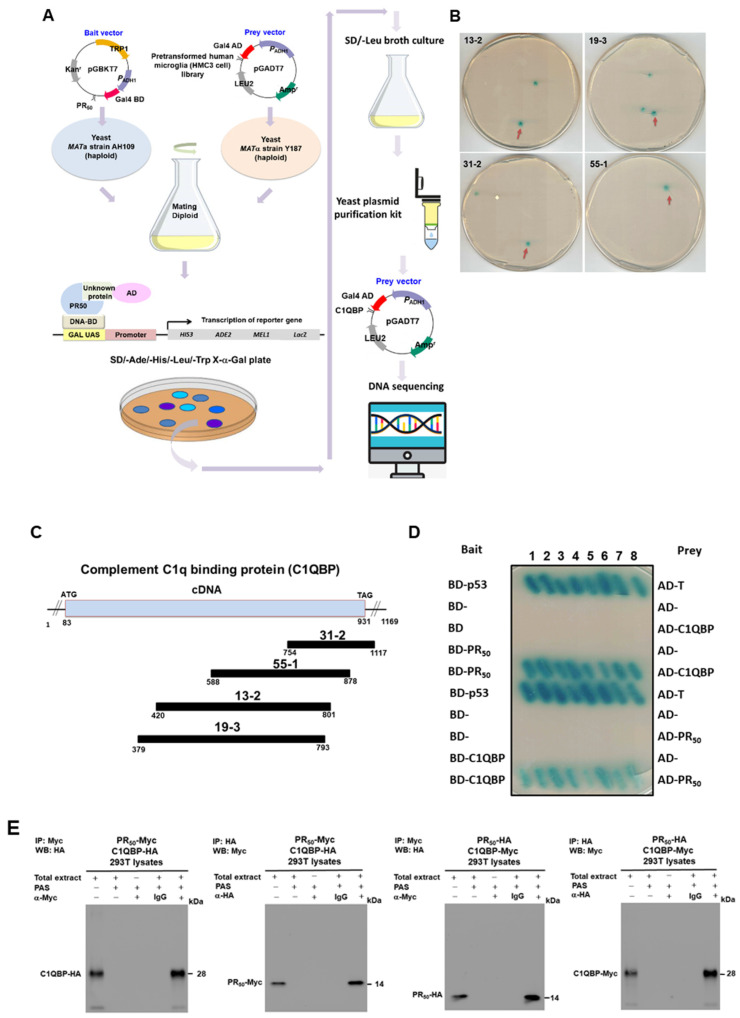

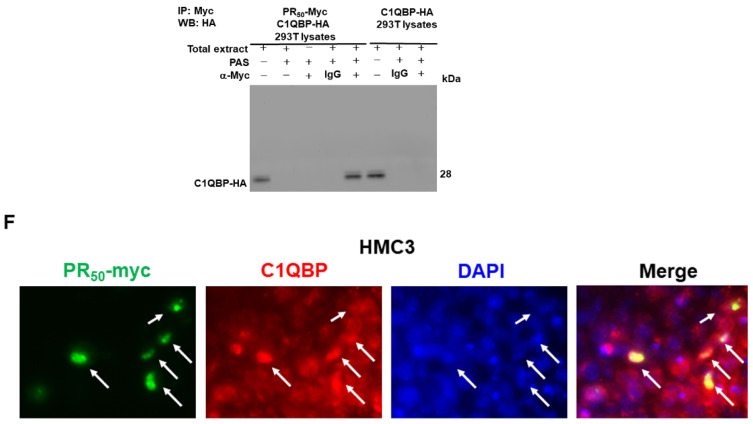
Complement component 1 q subcomponent-binding protein (C1QBP) is one of the specific interaction partners of the PR_50_ protein. (**A**) The flowchart displays the yeast two-hybrid strategy for screening human HMC3 microglial cells’ cDNA library using PR_50_ as bait. (**B**) Clones in the screening plate containing the C1QBP fragment are shown (red arrows). Blue diploid cells contain four reporter genes that are activated in response to two hybrid interactions. (**C**) Schematic drawing of the overlapping C1QBP cDNA clones that span the coding region of the C1QBP gene (NCBI reference sequence: NP_001203). C1QBP contains a mitochondrial glycoprotein MAM33-like domain. (**D**) Confirming the interaction between PR_50_ and full length C1QBP using yeast two-hybrid assay. Diploid cells containing BD-p53 and AD-T were used as a positive control. (**E**) PR_50_ interacts with C1QBP in an immune co-precipitation (co-IP) analysis. 293T cells were co-transfected with pCMV-PR_50_-myc and pCMV-C1QBP-HA. The cells were then lysed and co-immunoprecipitated with anti-myc antibody or anti-HA antibody, respectively. Finally, PR_50_ and C1QBP were identified on co-IP complexes with anti-HA antibody or anti-myc antibody by western blotting. Similarly, we co-transfected 293 T cells with pCMV-C1QBP-myc and pCMV-PR_50_-HA for co-IP. The cells were then lysed and co-immunoprecipitated with anti-HA antibody or anti-myc antibody, respectively. Finally, C1QBP and PR_50_ were identified on co-IP complexes with anti-myc antibody or anti-HA antibody by Western blotting. 293T cells were transfected with pCMV-C1QBP-HA as a negative control of co-IP. (**F**) PR_50_ (green) and C1QBP (red) are partially co-located in the nuclei of HMC3 cells by immunofluorescence staining analysis (white arrows). The nuclei were stained with DAPI.

To elucidate the potential mechanism of PR-DPR on the NLRP3 inflammasome activation in microglia as a possible strategy of therapeutic intervention in C9-ALS, we first wanted to identify factors that might interact with PR_50_ in HMC3 cells. We constructed a cDNA expression library of HMC3 cells for yeast two-hybrid screening (Figure 5A). As a result, 34 positive clones interacting with PR_50_ were obtained. After exclusion of the bait plasmid, followed by the extraction and sequencing of the prey plasmid, it was confirmed that four positive clones contained partial fragments of complement component 1 q subcomponent-binding protein (C1QBP, NCBI reference sequence: NP_001203) (Figure 5B,C).

We further performed a yeast two-hybrid analysis of PR_50_ with synthetic full-length C1QBP and then obtained positive clones (Figure 5D). Additionally, PR_50_ and C1QBP were fused with HA or myc tags and co-overexpressed in 293T cells. Then, the co-immunoprecipitation in 293 T cells was carried out. The results showed that both can be detected in the co-precipitated product (Figure 5E). In regards to immunofluorescence staining, we also observed that PR_50_ colocalized with part of C1QBP in the nucleus (Figure 5F).

**Figure 6:**
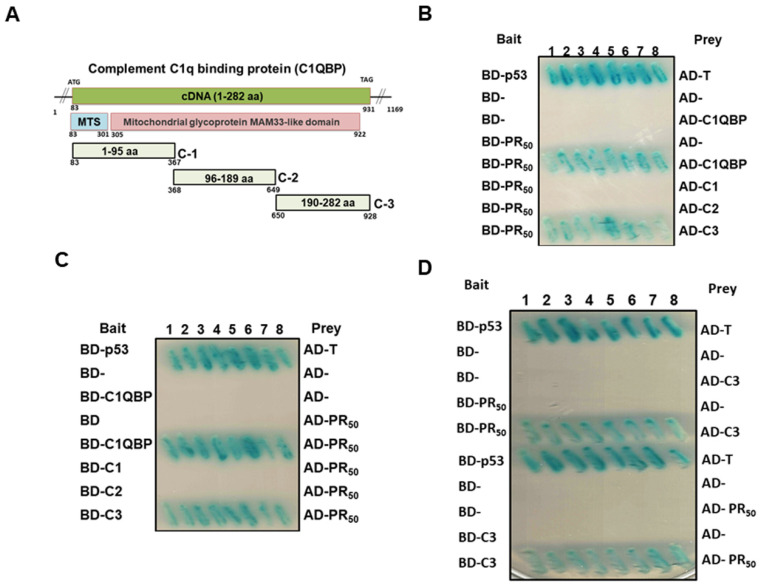
The identification of the region on the C1QBP that interacts with PR_50_ using yeast two-hybrid analysis. (**A**) Schematic drawing of the three C1QBP fragments used in yeast two-hybrid assays. (**B**–**D**) PR_50_ interacts exclusively with the C3 fragment (amino acid 190–282) of C1QBP in a yeast two-hybrid assay. Diploid cells (blue) contain four reporter genes that are activated in response to two hybrid interactions. Diploid cells containing BD-p53 and AD-T were used as a positive control.

Based on the above results, we identified C1QBP as one of the PR_50_ interaction partners. Next, we divided the amino acid sequence of C1QBP into three fragments, 1–95 (C1), 96–189 (C2) and 190–282 (C3), respectively, and performed a yeast two-hybrid analysis with PR_50_ to confirm the precise interaction area with PR_50_ (Figure 6A). As shown in Figure 6B–D, PR_50_ has a strong interaction with the fragment C3 of C1QBP.

### 3.5. Down-Regulation of C1QBP Expression Directly Caused NLRP3 Inflammasome Activation in Human HMC3 Microglial Cells and Covered the Effect of PR_50_ on NLRP3 Inflammasome Activation

**Figure 7:**
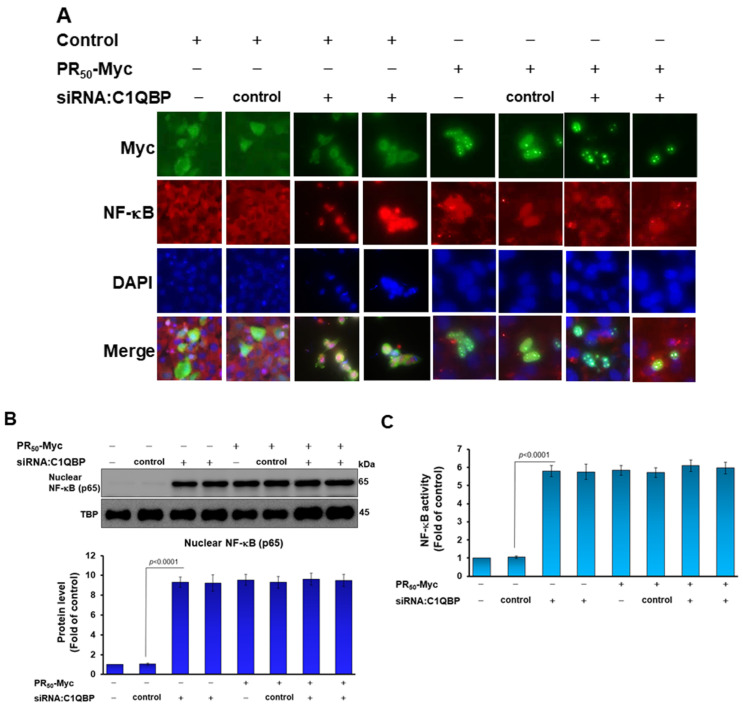

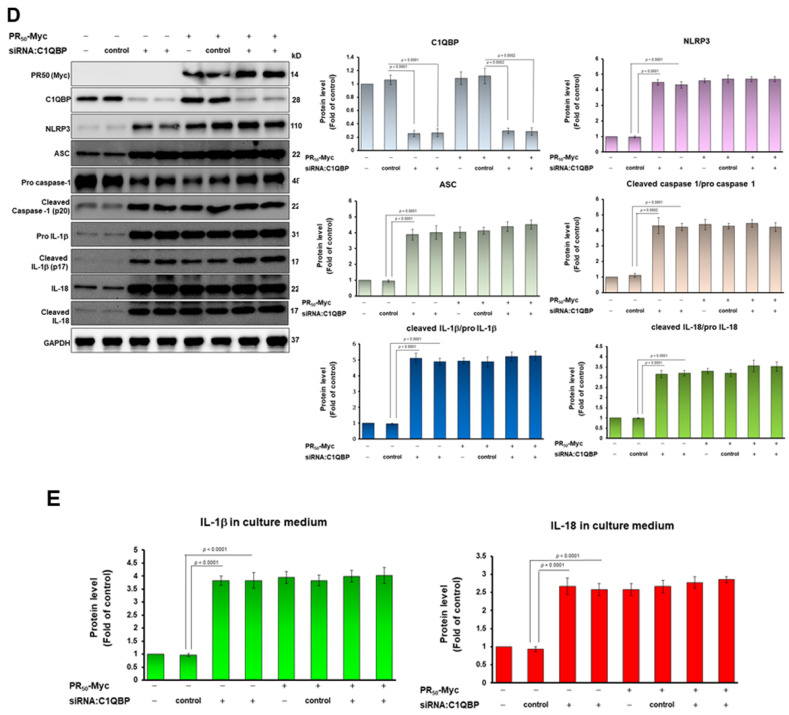
Knockdown of C1QBP directly caused NLRP3 inflammasome activation in HMC3 cells and covered the effect of PR_50_ on NLRP3 inflammasome activation. Control or PR_50_-expressing HMC3 cells were down-regulated the expression of C1QBP by RNAi for 24 h. (**A**) Immunofluorescence staining of NF-κBp65 (red) and Myc-tag (green). Knockdown of C1QBP promotes the movement of NF-κB p65 from the cytoplasm to the nucleus. DAPI was used for nuclear staining. (**B**) Extract of cell nucleus was analyzed for the level of NF-κB p65 by Western blotting. TBP was used as a loading control. The bottom panel shows quantitative analysis graphs using ImageJ software. (**C**) NF-κB p65 transcription factor activity assay showed that Knockdown of C1QBP promotes NF-κB activity. TBP was used as a loading control for input. (**D**) Cell lysates were analyzed for the expression of PR_50_, C1QBP, and NLRP3 inflammasome-related components by Western blotting. GAPDH was used as an internal loading control. The left panel is a representative set of images. The right panel shows a graph of quantitative analysis using ImageJ software. (**E**) The level of mature IL-1β and mature IL-18 in the conditional medium was measured by ELISA.

So far, no reports have suggested a direct relationship between C1QBP and NLRP3 inflammasome activation. Here, we wanted to explore the role of C1QBP in NLRP3 inflammasomes in HMC3 cells. We used RNAi to downregulate the expression of C1QBP in HMC3 cells. The result showed that knockdown of C1QBP can significantly induce the activity of NF-κB and NLRP3 inflammasomes in HMC3 cells. In the immunofluorescence staining and Western blotting, we found that the down-regulation of C1QBP promoted NF-κB (p65) to enter the nucleus compared with the siRNA control group (*p* < 0.0001) (Figure 7A,B). Similarly, RNAi of C1QBP augmented NF-κB (p65) activation compared with the siRNA control group (*p* < 0.0001) by NF-κB p65 transcription factor activity assay (Figure 7C). The Western blotting analysis indicated that the level of C1QBO in HMC3 cells treated with siRNA of C1QBP was only 24% (lane 3, *p* < 0.0001) compared with the control siRNA group (Figure 7D). Moreover, the expression of NLRP3 (lane 3, *p* < 0.0001), ASC (lane 3, *p* < 0.0001), cleaved caspase 1/pro caspase 1 (lane 3, *p* = 0.0002), cleaved IL-1β/pro IL-1β (lane 3, *p* < 0.0001), and cleaved IL-18/pro IL-18 (lane 3, *p* < 0.0001) related to the activity of the NLRP3 inflammasome was also significantly augmented compared with the control siRNA group (Figure 7D). The ELISA analysis also found that the level of mature IL-1β (lane 3, *p* < 0.0001) and mature IL-18 (lane 3, *p* = 0.0001) in the medium were significantly raised compared with the control siRNA group (Figure 7E).

Since the above results showed that the down-regulation of C1QBP can directly induce NLRP3 inflammasome activity, we wanted to confirm whether PR_50_ induces NLRP3 inflammasome activity by affecting the function of C1QBP in HMC3 cells. We treated PR_50_–expressing HMC3 cells with C1QBP siRNA for 24 h. In the immunofluorescence staining and Western blotting, we found that downregulation of C1QBP did not enhance the ability of PR_50_ to promote the entry of NF-κB (p65) into the nucleus (lanes 7 and 8, Figure 7A,B) and NF-κB (p65) activation (lanes 7 and 8, Figure 7C) compared with the control siRNA group. The results of Western blotting revealed that the level of C1QBO in PR_50_-expressing HMC3 cells was only 26% in the C1QBP siRNA group (lane 7, *p* = 0.0002) compared with the control RNAi group (Figure 7D). Additionally, in PR_50_-expressing C1QBP siRNA groups, the expressions of NLRP3, ASC, cleaved caspase 1 (p20), IL-1β, cleaved IL-1β (p17), IL-18 and cleaved IL-18 were not significantly changed compared with the PR_50_-expressing control siRNA group (lanes 7 and 8, Figure 7D). In ELISA, the levels of mature IL-1β and mature IL-18 in the conditional medium were not significantly changed in the PR_50_-expressing C1QBP siRNA group compared with the PR_50_-expressing control siRNA group (lanes 7 and 8, Figure 7E). The above results show that the down-regulation of C1QBP can directly induce NLRP3 inflammasome activity in HMC3 cells, which is similar to the NLRP3 inflammasome activity induced by PR_50_ expression. Furthermore, the PR_50_ expression did not further enhance the NLRP3 inflammasome activity induced by the C1QBP downregulation. It is speculated that PR_50_ may activate the NLRP3 inflammasome activity of HMC3 cells by binding and inhibiting the function of C1QBP.

### 3.6. Syringin (SRG) Prevents the Interaction of PR50 with C1QBP in a Yeast Two-Hybrid-Based Growth Assay

**Figure 8:**
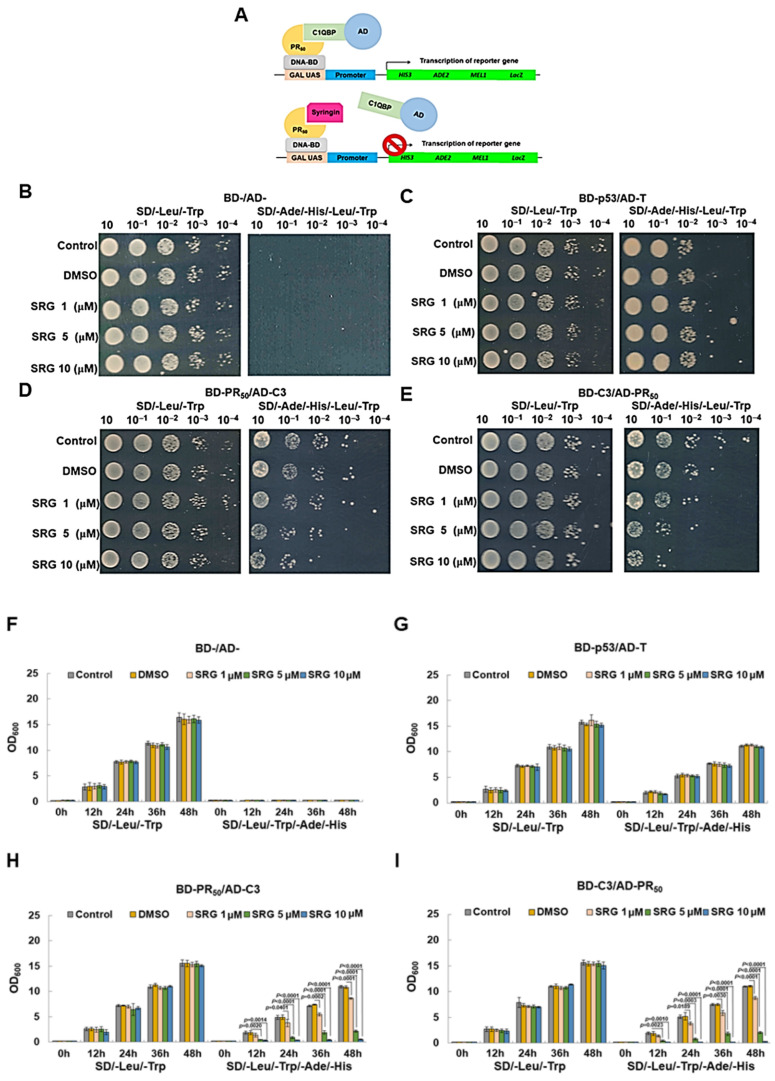
Syringin (SRG) blocked the interaction of PR_50_ and C1QBP in the yeast two-hybrid-based growth assay. (**A**) Schematic drawing the principle of the yeast two-hybrid-based growth assay in identifying inhibitors of PR_50_–C1QBP interactions. (**B**–**E**) Log-phase cultures of diploid yeast cells containing plasmids encoding either Gal4 BD-/Gal4 AD-, Gal4 BD-p53/Gal4 AD-T, Gal4 BD-PR_50_/Gal4 AD-C1QBP (C3) or Gal4 BD-C1QBP (C3)/Gal4 AD- PR_50_ were washed in water. We performed spot assays on non-selective (SD/-Leu/-Trp) and selective (SD/-Leu/-Trp/-Ade/-His) plates with serial dilutions of SRG and incubated them at 30 °C for 3 days. (**F**–**I**) Overnight cultures of diploid yeast cells containing plasmids encoding either Gal4 BD-/Gal4 AD-, Gal4 BD-p53/Gal4 AD-T, Gal4 BD-PR_50_/Gal4 AD-C1QBP (C3) or Gal4 BD-C1QBP (C3)/Gal4 AD-PR_50_ in a non-selective medium were washed in water and inoculated into non-selective and selective media at OD_600_ = 0.2 in triplicates to implement the optical density measures every 12 h for 2 days.

Since our study showed that the interaction of PR_50_ with C1QBP is the main cause of NLRP3 inflammasome activation in HMC3 cells, we further wanted to screen for small molecules that could disrupt this interaction using a yeast two hybrid-based growth assay [47]. The principle of this assay is that if a small molecule can enter the diploid yeast of the yeast two-hybrid system to block the interaction between PR_50_ and C1QBP, it will suppress the expression of the four reporter genes; as a result, that yeast cannot grow normally in the medium lacking adenine, histidine, Leucine, and tryptophan (Figure 8A). We screened and obtained a candidate-interfering molecule, syringin (SRG), using the small molecule library available in our laboratory. The growth of SRG-treated diploid yeast was quantified by spot assay and optical density (OD_600_) measurements, respectively. The results showed that diploid yeast of BD-/AD- treated with SRG below 10 μM on the SD/-Leu/-Trp culture medium had no significant effect on the growth. However, it did not grow in the SD/-Ade/-His/-Leu/-Trp medium, as expected (Figure 8B,F). The diploid yeast of BD-P53/AD-T under 10 μM SRG treatment did not significantly affect the growth of yeast in either the SD/-Leu/-Trp or SD/-Ade/-His/-Leu/-Trp culture media. However, the growth on SD/-Ade/-His/-Leu/-Trp was slightly slower (Figure 8C,G). This indicates that SRG treatment below 10 μM is not toxic to yeast. In the diploid yeast of BD-PR_50_/AD-C1QBP (C3), the growth of yeast was slightly slower than that of yeast diploid of BD-P53/AD-T without SRG treatment, which may be the outcome of PR_50_ toxicity. The growth of diploid yeast of BD-PR_50_/AD-C1QBP (C3) in the SD/-Leu/-Trp medium was also unaffected under 10 μM SRG treatment. However, the growth in SD/-Ade/-His/-Leu/-Trp culture medium showed a dose-dependent slowdown of SRG (*p* < 0.0001, 10 μM SRG, 48 h) (Figure 8D,H). This represents that the interaction of PR_50_ and C1QBP is attenuated by SRG treatment. The same result was also shown in the experiment with diploid yeast of BD-C1QBP (C3)/AD-PR_50_ (*p* < 0.0001, 10 μM SRG, 48 h) (Figure 8E,I).

### 3.7. Syringin (SRG) Prevents PR_50_-Induced NLRP3-Inflammasome Activity in HMC3 Cells and PR-CM-Caused Apoptosis in NSC-34 Cells

**Figure 9:**
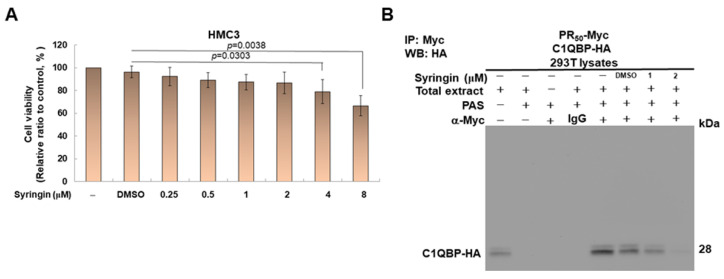

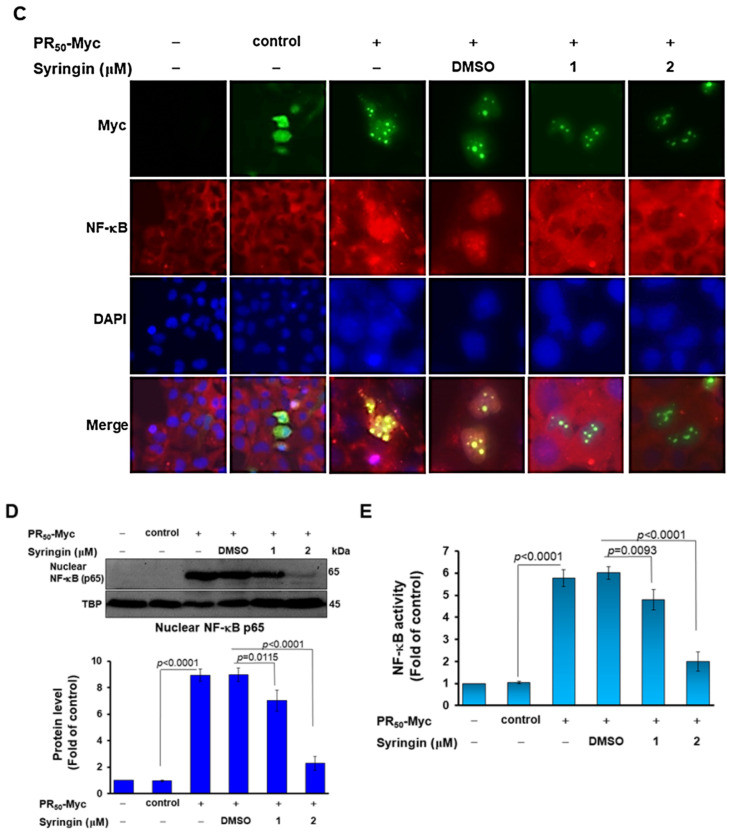

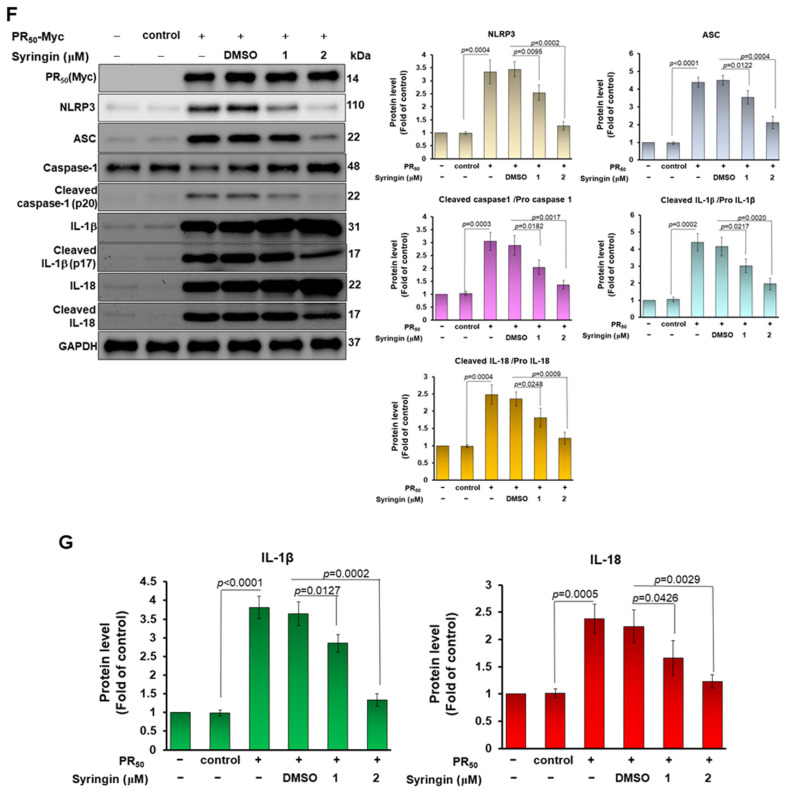

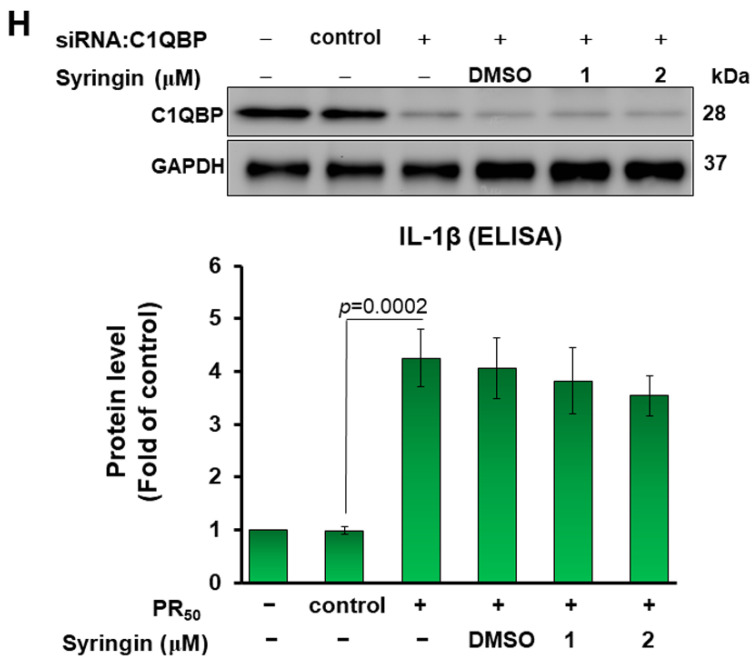
Syringin (SRG) inhibited NLRP3 inflammasome activity in PR_50_-expressing HMC3 microglial cell. (**A**) HMC3 cells were treated with 0.25, 0.5, 1, 2, 4, or 8 μM SRG for 24 h. Finally, the cell survival rate was determined by MTT assay. (**B**) Syringin (SRG) inhibited the interaction of PR_50_ and C1QBP in co-immunoprecipitation (co-IP) assays. 293T cells were co-transfected with pCMV-PR_50_-myc and pCMV-C1QBP-HA. The cells were then lysed, 1 or 2 μM SRG was added, and they were co-immunoprecipitated overnight with anti-myc antibody or anti-HA antibody, respectively. Finally, C1QBP and PR_50_ were identified on co-IP complexes with anti-HA antibody or anti-myc antibody by Western blotting. (**C**) Immunofluorescence staining of NF-κBp65 (red) and Myc-tag (green). PR_50_-expressing HMC3 cells were treated with 1or 2 μM SRG for 24 h. Treatment with SRG lessened the movement of NF-κB p65 from the cytoplasm to the nucleus. DAPI was used for nuclear staining. (**D**) Extract of cell nucleus was analyzed for the level of NF-κB p65 by Western blotting. TBP was used as a loading control. The bottom panel is a quantitative analysis graph using ImageJ software. (**E**) NF-κB p65 transcription factor activity assay showed that treatment of SRG diminished NF-κB activity. TBP was used as a loading control for input. (**F**) Cell lysates were analyzed for the expression of PR_50_ (Myc) and NLRP3 inflammasome-related components by Western blotting. GAPDH was used as an internal loading control. The left panel is a representative set of images, and the right panel shows quantitative analysis graphs using ImageJ software. (**G**) The level of mature IL-1β and mature IL-18 in the conditional medium were measured by ELISA. (**H**) Syringin (SRG) did not inhibit NLRP3 inflammasome activity in HMC3 microglial cells of C1QBP-knockdown. C1QBP siRNA-transfected HMC3 cells were treated with 1 or 2 μM SRG for 24 h. The level of C1QBP was analyzed by Western blotting. GAPDH was used as an internal loading control (Top panel). The level of mature IL-1β in the conditional media were measured by ELISA (bottom panel).

Since syringin (SRG) blocks PR_50_-C1QBP interaction in the yeast two-hybrid-based growth assay, we further evaluated the effect of this molecule on NLRP3-inflammasome activity in PR_50_-expressing HMC3 cells. First, we performed an MTT analysis to determine the appropriate concentration of SRG for treatment. The results showed that an SRG treatment below 2 μM for 24 h had no significant effect on the survival of HMC3 cells (Figure 9A). Next, we demonstrated that SRG dose-dependently reduced the interaction between PR_50_ and C1QBP in 293T cells using co-immunoprecipitation (Figure 9B). Moreover, SRG treatment for 24 h significantly dose-dependently lessened PR_50_-induced NLRP3 inflammasome activity in HMC3 cells. In the immunofluorescence staining, we found that the treatment of SRG diminished NF-κB (p65) to enter the nucleus (Figure 9C,D). Similarly, treatment of SRG lessened NF-κB (p65) activation compared with the untreated group (2 μM, *p* < 0.0001) by an NF-κB p65 transcription factor activity assay (Figure 9E). Using a Western blot quantification, the treatment with 2 μM SRG reduced the levels of NLRP3 (*p* = 0.0002), ASC (*p* = 0.0004), cleaved caspase 1 (p20)/pro caspase 1 (*p* = 0.0017), cleaved IL-1β (p17)/pro IL-1β (*p* = 0.0020), and cleaved IL-18/pro IL-18 (*p* = 0.0009) in PR50-expressing cells compared to the untreated group (Figure 9F). In the ELISA, we also found that the levels of mature IL-1β (*p* = 0.0002) and mature IL-18 (*p* = 0.0029) in the conditioned media of PR_50_-expressing HNC3 cells were significantly reduced compared to the untreated group (Figure 9G). The above data show that the activity of NLRP3 inflammasomes in microglial cells induced by intracellular PR_50_ can be inhibited by SRG. Notably, we also treated HMC3 cells of C1QBP-knockdown with SRG. The result showed that the activity of the activated NLRP3 inflammasome was not inhibited (Figure 9H).

**Figure 10:**
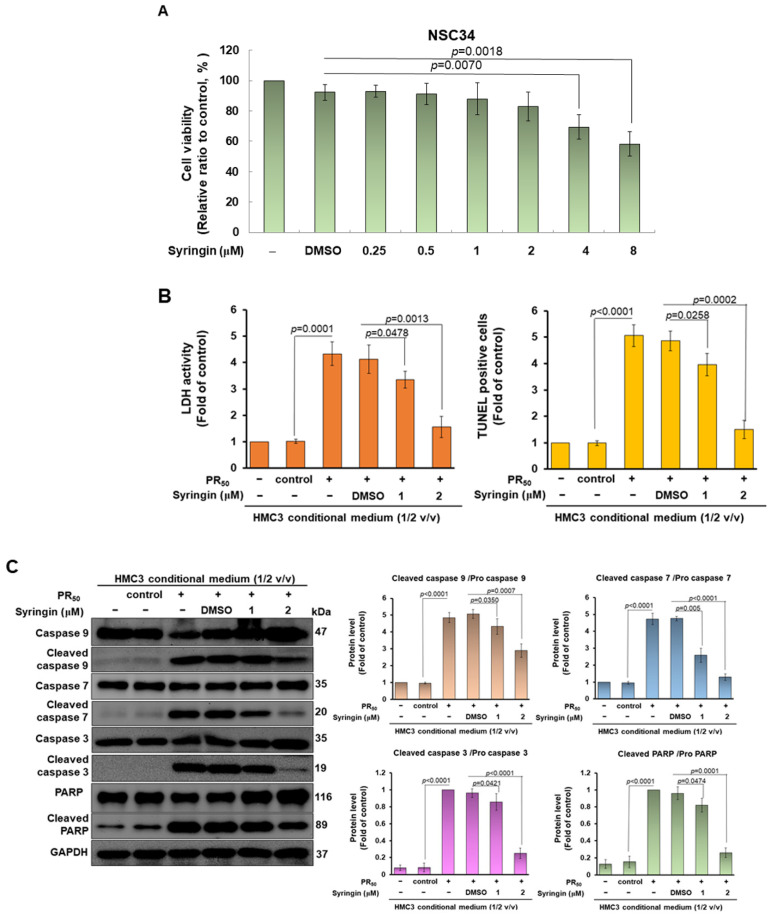
Treatment with syringin (SRG) abolished the ability that PR-CM caused apoptosis in mouse NSC-34 motor neuron cells. (**A**) NSC 34 cells were treated with 0.25, 0.5, 1, 2, 4, or 8 μM SRG for 24 h. Finally, the cell survival rate was determined by MTT assay. (**B**) NSC-34 cells were treated with SRG-treated or untreated PR-CM at 1/2 (*v*/*v*) ratios for 24 h. Cell death and apoptosis were measured by lactate dehydrogenase (LDH) release via ELISA and apoptotic-cell numbers via TUNEL assay. (**C**) Western blotting was used to quantify the levels of apoptotic core proteins (leaf panel). ImageJ software was used to obtain the signal intensity of the image (right panel). The loaded internal control is GAPDH.

Next, we wanted to determine whether SRG treatment could reverse motor neuron apoptosis induced by the conditioned media of PR_50_-expressing HMC3 microglia (PR-CM). We performed am MTT analysis to show that SRG below 2 μM for 24 h had no significant effect on the survival of NSC34 cells (Figure 10A). We then treated the NSC-34 cells for 24 h using SRG-treated or untreated PR-CM. The result showed that SRG diminished the ability of PR-CM to dose-dependently induce the release of lactate dehydrogenase (2 μM SRG, *p* = 0.0013) and the apoptotic cell number (2 μM SRG, *p* = 0.0002) in NSC34 cells (Figure 10B). We also found that the levels of cleaved caspase 9/pro caspase 9 (2 μM SRG, *p* = 0.0007), cleaved caspase 7/pro caspase 7 (2 μM SRG, *p* < 0.0001), cleaved caspase 3/pro caspase 3 (2 μM SRG, *p* < 0.0001) and cleaved PARP/pro PARP (2 μM SRG, *p* = 0.0001) also decreased in NSC-34 cells treated with the SRG-treated PR-CM compared with the SRG-untreated PR-CM by the Western blotting analysis (Figure 10C).

## 4. Discussion

C9-ALS has been shown to be closely related to the neuroinflammation of microglial cells [48]. However, the effect of dipeptide repeat proteins (DPRs), especially the most toxic proline-arginine (PR)-DPR, generated by C9-ALS on the NLRP3 inflammasome activity of microglial cells and their possible mechanism of action are still unclear. This study mainly explored the effect of PR-DPR on the activation of NLRP3 inflammasomes in microglial cells and related mechanisms. We demonstrated that the expression of PR-DPR (PR_50_) can induce the activity of NLRP3 inflammasomes in human HMC3 microglial cells, including the activation of NF-κB and the increase in the expression of ASC, NLRP3, cleaved caspase1, pro IL-1β, cleaved IL-1β, pro IL-18 and cleaved IL-18. Furthermore, mouse motor neuron NSC-34 cells release lactate dehydrogenase and increase apoptotic activity when treated with conditioned medium of PR_50_-expressing HMC3 cells (PR-CM). This suggests that motor neurons are degenerated and lost under the influence of NLRP3 inflammasome activity in the microglia. Moreover, the treatment with MCC950, an inhibitor of the NLRP3 inflammasome, reduced PR_50_-induced NLRP3 inflammasome activity in HMC3 cells, while avoiding the damage and apoptosis of NSC34 cells caused by PR-CM. Thus, this is the first report using a cellular model to demonstrate that endogenous PR-DPR in C9-ALS may activate NLRP3 inflammasome activity in microglia and affect motor neuron survival. However, PR-DPR may also activate NLRP3 inflammasome activity in microglia via other pathways. For example, PR-DPR expressed by neurons may be transferred extracellularly via exosomes, then bind to toll-like receptor 4 (TLR4) on the surface of microglia and activate the inflammasome activity, or activate downstream signaling pathways in microglial cells through endocytosis. However, this needs to be confirmed by further research in the future. We have shown that PR-DPR endogenous in microglia is not transported extracellularly.

To study the mechanism of PR-DPR-induced NLRP3 inflammasome activity, we used yeast two-hybrid screening, co-immunoprecipitation, and immunofluorescence staining, to confirm that complement component 1 q subcomponent-binding protein (C1QBP) is one of the interaction partners of PR-DPR. C1QBP is a highly conserved multifunctional protein mainly present in the mitochondrial matrix of animal cells. However, the forms in which the N-terminal mitochondrial targeting peptide is removed by proteolytic excision can also be found on the cell surface, cytoplasm, endoplasmic reticulum, nucleus, or extracellular space (exocrine). C1QBP has an asymmetric surface charge distribution. The negatively charged amino acid residues (mostly glutamate and aspartate) are mainly located on the soluble surface, while the membrane surface is mainly positively charged. This may explain how C1QBP binds to arginine-rich proteins, such as RAP80 containing an arginine-rich C-terminal domain or PR-DPR [49]. C1QBP can form homotrimers with a doughnut-like shape and the range of ligands for it is very broad [50,51,52,53]. C1QBP is involved in many key cellular processes. Those related to mitochondria include mitochondrial dynamics and metabolism, mitophagy, and oxidative phosphorylation (OXPHOS). Those associated with cancer include cell proliferation, migration, adhesion, and apoptosis. Immunologically, C1QBP is known to be involved in inflammation, autoimmunity, pathogen infection, wound coagulation, and healing. It also affects mRNA splicing during transcriptional regulation. Additionally, C1QBP is related to autophagy, nuclear-mitochondrial interaction, and cell signal transduction [52,54].

Many studies have shown that C1QBP is critical for mitochondrial biosynthesis, metabolism, and functional maintenance. C1QBP is involved in mitochondrial genome replication and regulates the activity of mitochondrial transcription factor A (TFAM), a key factor in packaging and maintaining mtDNA [55]. RECQ4 is a helicase involved in mt-DNA synthesis. C1QBP controls the transport of RECQ4 from the nucleus to the mitochondria to mediate mt-DNA synthesis [56]. The binding of C1QBP to mitochondrial RNase H1 affects mitochondrial pre-rRNA processing by increasing RNAse H1 hydrolytic activity, reducing the enzyme’s affinity for heteroduplex substrates and increasing turnover [57]. C1QBP is also a chaperone protein for RNAs and proteins involved in mitochondrial translation and function. Notably, RAP80, a nuclear DNA damage repair protein, interacts with C1QBP in mitochondria, thereby affecting mitochondrial protein biosynthesis [49]. Additionally, C1QBP can bind to components of the pyruvate dehydrogenase (PDH) complex located in the mitochondrial matrix, thereby regulating the synthesis of acetyl-CoA in the TCA cycle, and finally affecting ATP production.

C1QBP is also involved in mitochondrial quality control. Parkin is a protein involved in mitochondrial fission and mitophagy in neurons. C1QBP regulates mitochondrial morphology and dynamics by promoting parkin-mediated mitophagy. The depletion of C1QBP results in mitochondrial fragmentation [58]. Moreover, C1QBP negatively regulates the rate of degradation of the mitochondrial protein smARF, thus promoting smARF-induced autophagy and mitochondrial membrane potential dispersion [59]. Similarly, C1QBP can affect the enhancement of autophagic flux and the degradation of damaged mitochondria under starvation by regulating the stability and kinase activity of Unc-51-like kinase-1 (Ulk1) [60]. C1QBP is also involved in the OMA1-dependent proteolytic processing of the tooptic atrophy type 1 GTPase protein (OPA1), which is responsible for mitochondrial inner membrane fusion. This protective inhibition avoids cellular stress caused by mitochondrial fragmentation and swelling [61]. Furthermore, C1QBP depletion downregulates the levels of the mitochondrial outer membrane fusion proteins mitofusin 1 (Mfn1) and mitofusin 2 (Mfn2), resulting in abnormal mitochondrial structure and fragmentation, and ultimately in decreased OXPHOS [62]. Interestingly, C1QBP regulates the formation of the mitochondrial permeability transition pore (MPT) that cyclophilin D participates in. C1QBP mediates the inhibition of MPT formation in cells to assist cells against oxidative stress-induced mitochondria-dependent apoptosis [63,64]. C1QBP has also been shown to play an important role in Ca^2+^ cellular translocation and positively regulates Ca^2+^ uptake in the mitochondrial matrix, thereby regulating the activity of OXPHOS and apoptosis [65,66].

The defects of mitochondria can trigger inflammasome activation. For example, the release of mitochondrial reactive oxygen species (mtROS) is an important key upstream event of NLRP3 activation. The unbalanced regulation of mitochondrial fusion and fission results in mtROS adversity and OXPHOS deficiency, promoting NLRP3 inflammasome activation [67]. Maintaining mitochondrial functional integrity to reduce mtROS production effectively inhibits NLRP3 inflammasome activation [34]. Additionally, mtDNA dissociated from the mitochondria to the cytoplasm also causes the activity of the NLRP3 inflammasomes [68]. Under oxidative stress, mtDNA cytoplasmic escape was observed in the mitochondrial permeability transition pore (mPTP) in nucleus pulposus cells, triggering cGAS-STING-NLRP3 axis-dependent pyroptosis [69]. In brown adipose tissue, the loss of the mitochondrial reactive oxygen species scavenging protein thioredoxin 2 (TRX2) induces excess mtROS, leading to the disruption of mitochondrial integrity and the cytoplasmic release of mtDNA. Finally, mtDNA initiates the cGAS-STING and NLRP3 inflammasome pathways [70]. Furthermore, the mitophagy-mediated maintenance of mitochondrial homeostasis may limit NLRP3 inflammasome hyperactivation and its consequences in various neurological diseases [71]. Taken together, the dysfunction of C1QBP can disrupt the mitochondria, leading to mtROS production and mtDNA escape. These may ultimately induce NLRP3 inflammasome activity. Notably, some viral responses prevent apoptosis by reducing the C1QBP translocation to the mitochondria and promoting C1QBP formation into cytoplasmic complexes [72]. In mouse RAW264.7 macrophages, C1QBP leaks from the mitochondria into the cytoplasm during DNA virus infection and binds to the DNA sensor cyclic GMP-AMP (cGAMP) synthase (cGAS) to inhibit NLRP3 inflammasome activity. This promotes viral infection by preventing the inflammasome activity associated with the innate immune response elicited by cytoplasmic viral DNA [73]. This suggests that C1QBP has the ability to inhibit inflammasome activity. A study showed that C1QBP expression increases when microglial cells are subjected to stress [54]. Additionally, C1QBP is involved in the function of the endoplasmic reticulum and lysosome. Its functional inhibition causes endoplasmic reticulum stress [74] and lysosomal destruction [75] and induces NLRP3 inflammasome activity [76].

**Figure 11:**
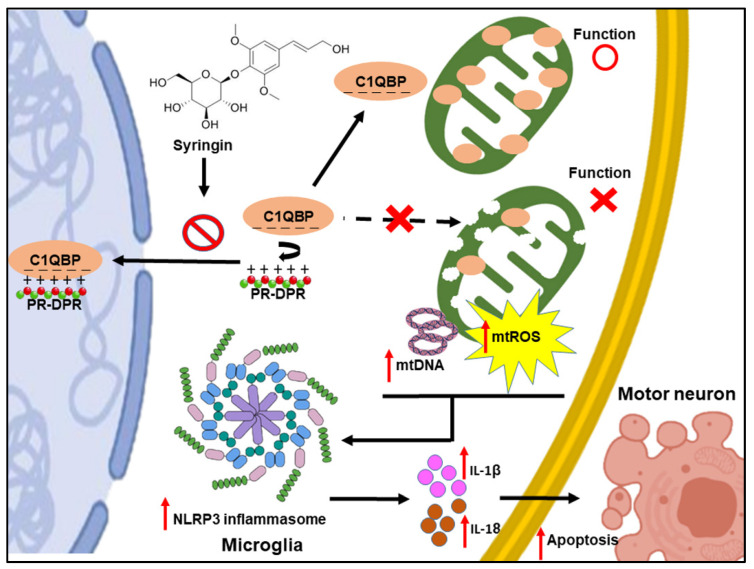
PR_50_ binds C1QBP and inhibits its function, resulting in mtROS production and mtDNA escape. Finally, NLRP3 inflammasome activity in microglia is induced. Syringin (SRG) treatment reduced the interaction between C1QBP and PR_50_, and hence attenuated PR_50_-induced NLRP3 inflammasome activity in microglia as well as PR-CM-caused apoptosis in motor neuron cell.

There are no reports demonstrating that C1QBP deficiency directly affects NLRP3 inflammasome activity. In this study, we found that the knockdown of C1QBP in human HMC3 microglia significantly induced the NLRP3 inflammasome activity at a level similar to that induced by PR_50_ expression, and its conditioned medium also induced the apoptosis of mouse motor neuron NSC-34 cells. Moreover, no cumulative additive effect of NLRP3 inflammasome activity was observed in HMC3 cells expressing PR_50_ and the knockdown of C1QBP, indicating that PR_50_ and C1QBP may be located on the same activation pathway of the NLRP3 inflammasome. We also found that PR_50_ can significantly increase the accumulation of C1QBP in the nucleus. This reveals that PR_50_ disrupts the mitochondrial translocation of C1QBP, ultimately leading to an aberrant reduction in mitochondrial C1QBP. These results suggest that PR_50_ binds to C1QBP and inhibits its function, resulting in mtROS production and mtDNA escape. Finally, the NLRP3 inflammasome activity in microglia is induced (Figure 11). Recent studies have shown that the ALS-associated mitochondrial protein CHCHD10 interacts with C1QBP [77]. Whether PR-DPR competitively binds to C1QBP and inhibits the function of CHCHD10, leading to ALS, deserves further study.

According to our study, inhibiting the interaction between PR-DPR and C1QBP in microglial cells may be a potential therapeutic strategy to deal with PR-DPR-induced NLRP3 inflammasome activity. We used a yeast two-hybrid-based growth assay and co-immunoprecipitation to screen our laboratory’s small compound library and found that a treatment with syringin (SRG) reduced the interaction between C1QBP and PR_50_. SRG treatment attenuated the PR_50_-induced NLRP3 inflammasome activity in microglia and hence improved PR-CM-caused motor neuron death and apoptosis (Figure 11). However, SRG did not inhibit the NLRP3 inflammasome activity induced by C1QBP knockdown. This further confirms its specific role in interfering with the interaction of PR_50_ and C1QBP. Natural bioactive glucoside syringin is purified from the root of *Acanthopanax senticosus* (Rupr. Maxim.). It has significant anti-inflammatory and neuroprotective properties [78,79]. It also prevents multiple NF-KB-signaling pathways to improve inflammatory diseases, such as asthma [80,81]. Furthermore, SRG protects cells from Aβ-induced toxicity and reduces neuronal ROS and apoptosis [82]. In a model of Alzheimer’s disease, the SRG-mediated enhancement of the interaction between the inhibitor of apoptosis-stimulating protein of p53 (iASPP) and Keap1 upregulates Nrf2 activity and leads to increased synaptic plasticity and the rescue of cognitive impairment [83]. Therefore, SRG inhibited the PR_50_-induced NLRP3 inflammasome activity in microglia as well as provided neuroprotective effects on NF-κB-related inflammation, mitochondrial dysfunction, and apoptosis. Here we propose that SRG can be regarded as a candidate drug for treating C9-ALS. Notably, the treatment of C9-ALS with general NLRP3 inflammasome inhibitors may diminish the overall innate immune response of the host. However, the use of SRG, a specific inhibitor of the interaction of PR_50_ with C1QBP, avoids this problem. 

Finally, our study showed that a conditional medium of PR_50_-expressing HMC3 cells induced apoptosis in mouse motor neuron NSC-34 cells. This may be induced by IL-1β and IL-18, the products of NLRP3 inflammasome activity [84]. Previous studies have revealed that microglia-derived IL-1β promotes neuronal apoptosis through the ER stress-mediated PERK/eIF2α/ATF4/CHOP-signaling pathway [85] or the activation of p38 mitogen-activated protein kinase [86]. In the mouse model of fibrillar α-synuclein of Parkinson’s disease, the NLRP3 inflammasome of microglia is activated, resulting in the extracellular release of IL-1β. The oral administration of the small molecule NLRP3 inhibitor MCC950 in mice inhibits inflammasome activation and effectively relieves Parkinson’s disease-related motor dysfunction, nigrostriatal dopaminergic degeneration, and α-synuclein aggregates [87]. Therefore, improving IL-1β-induced neuronal damage and apoptosis is also a therapeutic strategy for C9-ALS.

## 5. Conclusions

In this study, we confirmed that PR-DPR induces the activity of NLRP3 inflammasomes in microglial cells by blocking C1QBP function and that the conditional medium of PR-DPR-expressed microglia causes the apoptosis of motor neurons. Additionally, syringin (SRG) prevents the interaction of PR_50_ with C1QBP, thereby diminishing the NLRP3 inflammasome activity in PR_50_-expressing microglia and then improving the apoptosis of motor neurons caused by NLRP3 inflammasome activity. Our results derived from this study clarify the roles of PR-DPR and C1QBP in the NLRP3 inflammasome activation of microglia of C9-ALS. We believe that these results provide not only a new concept for investigating the mechanism of C9-ALS but also possible strategies for C9-ALS therapies.

## Data Availability

All data used and analyzed during the current study are available from the corresponding author upon reasonable request.
